# Supplementary material for: An Evolutionary Conservation and Druggability Analysis of Enzymes Belonging to the Bacterial Shikimate Pathway
Source: Antibiotics (Basel). 2022 May 17;11(5):675. doi: 10.3390/antibiotics11050675 (PMC9137983; doi:10.3390/antibiotics11050675)
Supplement: Supplementary file 1 [file antibiotics-11-00675-s001.zip › antibiotics-1713583-supplementary.pdf]

SUPPORTING INFORMATION

for

Evolutionary Conservation and Druggability Analysis of Enzymes Belonging to the Bacterial Shikimate Pathway

Rok Frlan*

Corresponding author address: University of Ljubljana, Faculty of Pharmacy, 1000 Ljubljana, Tel: +386 1 4769 674, e-mail: rok.frlan@ffa.uni-lj.si

1. **General data** S2
2. **General procedures** S4
   2.1. Multiple sequence analysis S4
   2.2. Comparisons of lengths between isozymes S5
   2.3. Analysis od druggability S5
      2.3.1. Acquisition and preparation of proteins in Maestro S5
      1.3.2. Identification of hotspots and binding sites with FTMap and FTSite S5
      1.3.3. Identification of binding sites and the assessment of druggability using SiteMap S6
   1.4. Generation of figures of heatmaps and binding sites S6
2. **Experimental Data** S6
   2.1. Multiple sequence analysis S6
   2.2. DAHPsynthase S7
      2.2.1. Length analysis S7
      2.2.2. Conservation analysis by ConSurf S7
      2.2.3. Heatmaps and binding sites detected by FTMap and Sitemap S8
   2.3. DHQS S8
      2.3.1. Conservation analysis by ConSurf S8
   2.3.2. Heatmaps and binding sites detected by FTMap and Sitemap S9
   2.4. DHQase S10
      2.4.1. Length analysis S10
      2.4.2. Type I S10

2.4.3. Type II S12
2.5. SDH S13
2.5.1. Conservation analysis by ConSurf S13
2.5.2. Heatmaps and binding sites detected by FTMap and Sitemap S14
2.6. SK S15
2.6.1. Analysis of length S15
2.6.2. Conservation analysis by ConSurf S15
2.6.3. Heatmaps and binding sites detected by FTMap and Sitemap S16
2.6.4. Conservation analysis by ConSurf S17
2.6.5. Heatmaps and binding sites detected by FTMap and Sitemap S18
2.7. EPSPS S18
2.7.1. Conservation analysis by ConSurf S18
2.7.2. Heatmaps and binding sites detected by FTMap and Sitemap S19
2.8. CS S20
2.8.1. Conservation analysis by ConSurf S20
2.8.2. Heatmaps and binding site analysis S21
2.9. Druggability analyses S23
2.9.1. Data S23
2.9.2. Graphical representation of druggability analyses S31
2.10. Analyses of physicochemical properties S33
3. References S34

1. General data

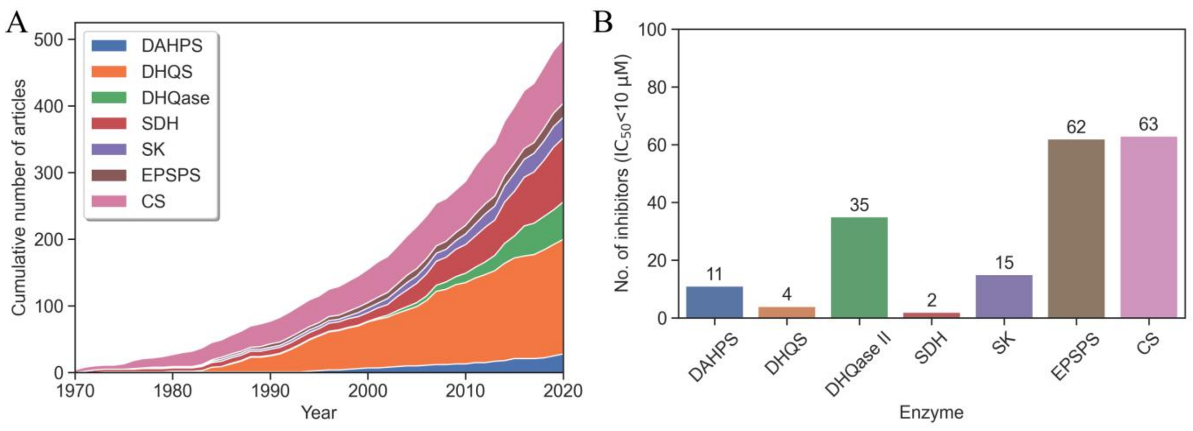

Figure S1. The number of inhibitors of the shikimate pathway enzymes. (**a**) a cumulative number of articles on shikimate pathway inhibitors per each enzyme published from 1970-2020; (**b**) The number of low micro and submicromolar inhibitors of each enzyme. Enzyme abbreviations: 3-deoxy-*D*-arabino-heptulosonate-7-

phosphate-synthase (DAHPS), 3-dehydroquinate (DHQ) synthase (DHQS), 3-dehydroquinate dehydratase (DHQase), shikimate 5-dehydrogenase (SDH), shikimate kinase (SK), 5-enolpyruvylshikimate-3-phosphate (EPSP) synthase (EPSPS), chorismate synthase (CS).

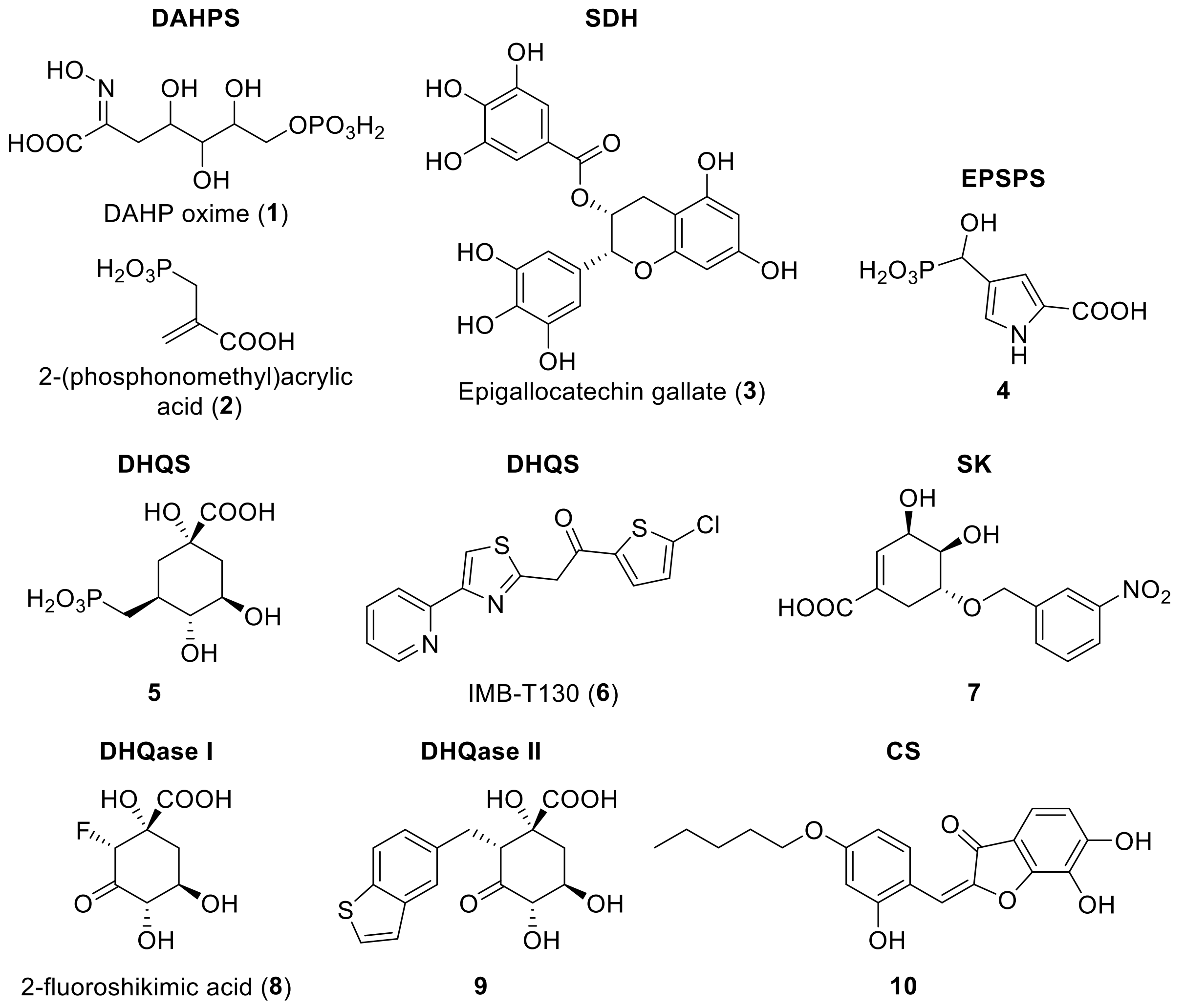

Figure S2. Inhibitors **1**-**10** of bacterial enzymes belonging to the shikimate pathway [1–11].

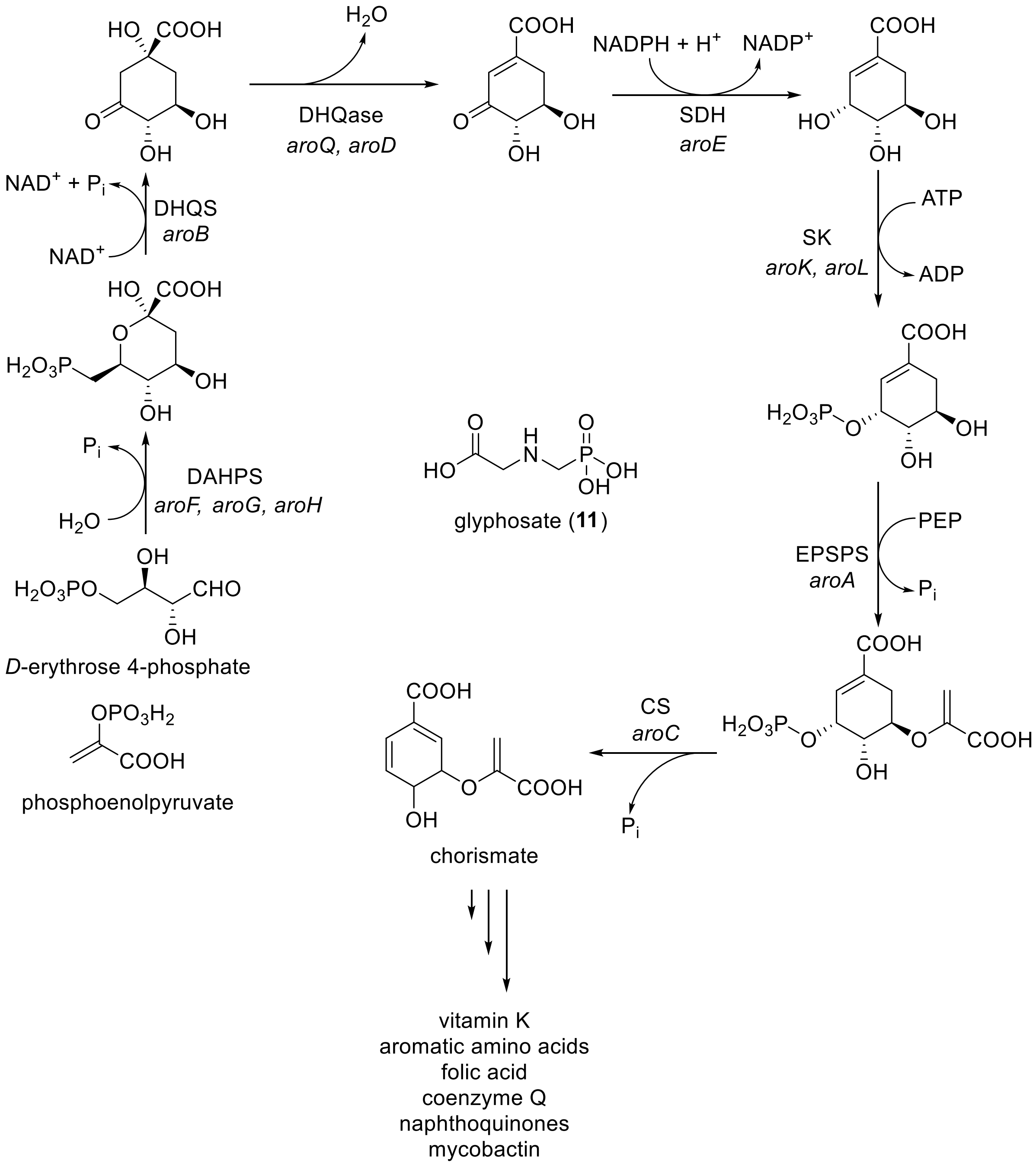

Figure S3. Biosynthetic steps of the shikimate metabolic pathway [12–16].

2. General procedures

2.1. Multiple sequence analysis

The three enzymes in this pathway, namely DAHPS, DHQase, and SK, each exist in at least two isozymes that differ in their physicochemical properties, amino acid sequences, quaternary structures, and molecular mass. Details of the exact length of each isozyme can be found in Table S2, Figure S4, and Figure S5. DAHPS are classified into three major groups: Type Iα, Type Iβ, or Type II. Although there is less than 10% sequence identity between type I and type II classes [17–22], both types share a common 3D structure. Because 14% of the analyzed genomes harbored both isozymes, both sequences

were used in the MSA. DHQases are also divided into two groups, type I and type II, showing no clear similarity in amino acid sequence [23,24] and little similarity in the three-dimensional structures of both enzyme families, as evidenced by a large average root mean square deviation (RMSD = 11.47 ± 1.10Å) of the superimposed crystal structures. In contrast, there is a large structural similarity within both families with an RMSD of 0.67 ± 0.21 Å and 0.61 ± 0.09 Å for families I and II, respectively. Because of these large differences, the two families were analyzed separately as single units. SKs are divided into two groups: type I and type II. SK II is the dominant enzyme that has a hundredfold higher affinity (KM=200μM) for shikimate than the enzyme SK I (KM = 20 mM). In 20% of pathogenic bacterial species, for example in *E. coli*, *K. pneumoniae*, *Streptococcus sp.* and *Y. pestis*, both isozymes are present. The role of SK I is not yet clear, but it has been suggested that it has an alternative biological function related to sensitivity to the antibiotic mecillinam [25]. Therefore, only SK II was used for further analysis.

2.2. Comparisons of lengths between isozymes

Protein sequences of individual enzymes from all species were downloaded from the UniProt database [26]. Outliers that had only 60% residues compared with the average length of the protein family were filtered out and then a unique representative was selected for each human pathogenic bacterium. A list of the number of human pathogenic microorganisms was obtained from the Kyoto Encyclopaedia of Genes and Genomes [27]. A comparison of protein lengths between the isozymes of DAHPS, DHQase, and SK as measured by the number of amino acids is shown in Table S2, Figure S4, and Figure S5.

2.3. Analysis od druggability

2.3.1. Acquisition and preparation of proteins in Maestro

Selected 3D structures were imported from the Protein Data Bank into Maestro [28]. To prevent regions between protein monomers from forming unphysical sites that give good results but exist only in the crystal lattice and not in solution, only one monomer chain was retained, usually chain A. Structures were first aligned using Protein Structure Alignment and then prepared using Protein Preparation Wizard both implemented in the Schrödinger Suite [28]. In each structure, ligands, water, and other co-crystallized molecules were removed except for the Mg cofactor if present. Bond orders were automatically assigned, hydrogens were added, selenomethionines were converted to methionines, missing side chains were added, ligands were removed, disulfide bridges were created when possible, water beyond 5Å radius of heteroatoms was added, and heteroatoms were protonated at pH 7.0. The impred script was used to perform constrained minimization of the protein with a maximum root mean square deviation (RMDS) of 0.30Å.

1.3.2. Identification of hotspots and binding sites with FTMap and FTSite

Representative PDB structures were uploaded to the FTMap and FTSite [29] servers for mapping. Subsequently, the results were downloaded as a Pymol [30] session file(.pse) for graphical representation and further analysis. Amino acids within 4Å of each cluster were selected and exported in pdb format. PDBest [31], a freely available platform for biomolecule manipulation, filtering, and normalization was used to extract residue numbers that were further

processed using KNIME [32]. Typical examples of analyses for one representative bacteria per enzyme are provided in Tables S3-S29.

1.3.3. Identification of binding sites and the assessment of druggability using SiteMap

All protein structures were first prepared according to the procedure described above. Then SiteMap [33], the binding site identification software, was applied to all selected enzymes. The potential binding sites with the highest score were identified. Sites were retained if they comprised at least 15 site points per reported site. The narrower definition of hydrophobicity was used along with a fine grid (0.35 Å). Site maps that were 4Å or more from the nearest site points were truncated. Upon completion, the results were downloaded as csv file and then further analyzed using KNIME [32], extracting physicochemical property data and residue numbers for further processing. The graphical representations of the physicochemical properties, shown in Figures 6 and 7 were generated from data in Table S30.

1.4. Generation of figures of heatmaps and binding sites

Graphical representation of the three-dimensional structures of proteins with heatmaps and binding sites mapped onto the protein structure generated by FTMap and FTSite server [29] was performed using Pymol [30]. All session files downloaded from FTMap and FTSite server [29] were combined into a common session file. The structures of each enzyme were first aligned using the align command in PyMol. The three highest-rank heatmaps and binding sites were selected and then plotted as a surface representation on a representative X-ray structure and presented in Figures S6 and S7. A graphical representation of the SiteMap results was performed in Maestro. The results of the analyses for six representative structures per enzyme were superimposed and then two sites with the highest rank by DScore were selected and plotted on a representative X-ray structure shown in Figures S6 and S7.

Boxplots were generated using the Seaborn [34] and Matplotlib [35] libraries in the Jupyter notebook [36].

2. Experimental Data

2.1. Multiple sequence analysis

Table S1. Sequences that were extracted from UniProt database.

Enzyme	Total number	Bacteria (%)	Fungi (%)	Plants (%)	Other (%) [1]	Analyzed [2]
DAHPS	52205	89.0	6.8	2.5	1.7	240
DHQS	39880	93.1	2.8	0.2	3.9	204
DHQase	45628	87.7	6.9	2.0	3.4	223
SDH	68219	91.4	4.8	1.5	2.3	205
SK	45865	89.7	4.7	2.0	3.6	201
EPSPS	37944	94.3	2.6	0.1	3.0	209
CS	38794	91.0	2.8	1.3	4.9	207

[1] Algea, archeae and apicomplexan parasites, [2] number of sequences from different pathogenic bacteria that were used for the analysis.

2.2. *DAHPsynthase*

2.2.1. Length analysis

Table S2. Average length and mass of DAHPS isoforms together with the number of sequences from different pathogenic bacteria found in Uniprot database.

DAHPS family	Isoform	Sequence length (AA)	Protein mass (kDa)	No. sequences
Type I	Aro F	355±7	39.1±0.7	64
	Aro G	353±6	38.7±0.6	88
	AroH	349±2	38.6±0.3	29
Type II	Aro F	448±2	50.2±0.4	2
	Aro G	460±7	50.8±0.6	31
	AroH	457±10	51.1±1.1	14

2.2.2. Conservation analysis by ConSurf

Table S3. Highly conserved residues (conservation score 9), conserved residues (conservation score 8), and variable residues (conservation scores 1–3) of DAHPS from *E. coli* (PDB: 1GG1), identified by ConSurf server [37]. Residues in bold print show no variation.

Residue classification	Residues
Highly conserved (Score = 9)	D51, R53, G59, P60, **C61**, S62, I63, A69, M91, R92, V93, Y94, E96, K97, P98, R99, T100, K105, **G106**, N109, G122, S158, A161, I162, **G163**, **R165**, T166, **H172**, S177, G184, **K186**, T189, H207, F209, G216, T223, **G225**, N226, L233, R234, **N240**, **D265**, S267, **H268**, **N270**, S271, K273, Q288, G298, M300, **E302**, S303, G308, Q310, A319, K322, I324
Conserved (Score = 8)	I46, H64, V90, G103, W104, D110, L129, P139, A141, D155, L156, A164, S169, N187, A196, S211, G236, Y241, Q278, V281, P317, G321
Variable (Score = 1-3)	D121, G257, S311, D333, D85, K128, A290, V345, E12, D52, K70, C286, D335, A344, K11, I13, K14, E15, L17, E24, K25, F26, E30, N31, N34, A37, H38, K41, K45, K48, N50, V67, T74, A78, E81, H112, N115, F117, P206, W215, G227, E238, K244, A247, E248, E251, G252, N254, P259, D280, A283, Q287, G291, K294, L312, E313, C328, E332, A336, R339, A342, N343

Table S4. Amino acids of PEP, E4P and allosteric binding sites of DAHPS from *E. coli* (PDB: 1GG1), with their ConSurf scores and maximum identity.

PEP binding site			E4P binding site			Allosteric site		
Residue	Score	Max identity (%)	Residue	Score	Max identity (%)	Residue	Score	Max identity (%)
R92	9	77.5	K97	9	99.6	M147	4	40.8
Y94	9	77.5	P98	9	99.6	P150	4	51.3
K97	9	99.6	R99	9	99.2	Q151	4	65.8
G163	9	100	T100	9	77.5	A154	7	46.7
A164	8	77,5	D326	4	34.3	L175	7	37.1
R165	9	100				G178	4	64.6
K186	9	100				L179	7	67.1
R234	9	78.5				S180	7	64.6
H268	9	100				S211	8	52.7

E302	9	100	V212	4	28.5

2.2.3. Heatmaps and binding sites detected by FTMap and Sitemap

Table S5. Amino acid residues and the corresponding clusters of DAHPS from *E. coli* (PDB: 1GG1) identified by FTMap and SiteMap.[37] Conserved and highly conserved residues are in bold (scores 8 and 9).

Heatmaps	Residues	Site	Residues
F1	**C61**, **K97**, **P98**, **R99**, T325, D326	S1	R40, P150, Q151, A154, L175, G178, L179, S180, C181, **F209**, **S211**, V212, T213, V221, **T223**
F2	**R92**, **Y94**, **K97**, E143, **A161**, **G163**, **A164**, **R165**, **K186**, **R234**, **H268**	S2	**R92**, **Y94**, **K97**, **P98**, **R99**, **T100**, E143, **G163**, **A164**, **R165**, **K186**, **R234**, **H268**, T325, D326
F3	I148, P150, Q151, E174, L175, A176, **S177**, G178, L179, **F209**, **S211**, V212, V221, **T223**	S3	K14, E15, L16, I108, F117, I119, N120, L123, D146, I148, T149, Y152
F4	Q151, A154, G178, L179, S180	S4	**I46**, L47, G49, **D51**, D52, **R53**, L54, P259, Q261, K294, A295, I296, I297
F5	L23, R127, P150, Q151, Y152, L153, A154, **D155**, K214	S5	H44, L47, K48, I200, G227, D228, C229, L258, P259, Q261
F6	I148, P150, Q151, E174, L175, A176, **S177**, G178, L179, **F209**, **S211**, V212, V221, **T223**	S6	F95, **E96**, I108, G142, E143, F144, L145, D146, T149, P150, L153, A154, M157
F7	F95, **E96**, **K97**, **K105**, **G106**, I108, **N109**, I119, L145, D146, T149		
F8	**E96**, I108, E143, F144, L145, D146, T149, P150, L153, A154		
F9	**V93**, **Y94**, F95, **E96**, G142, E143, F144, L153		
F10	A176, **S177**, G178, L179, S180, C181, P182, S224, **G225**, **N226**, G227, D228		
F11	R40, H44, L179, S180, C181, P182, **N226**, D228		
F12	A260, Q261, K294		
F13	K14, E15, L16, I119, N120		
F14	**G236**, K237, E238, A269, S272		

2.3. DHQS

2.3.1. Conservation analysis by ConSurf

Table S6. Highly conserved residues (conservation score 9), conserved residues (conservation score 8) and variable residues (conservation scores 1–3) of DHQS from *A. baumannii* (PDB: 5EKS) identified by ConSurf server [37]. Residues in bold print show no variation.

Residue classification	Residues
Highly conserved (Score = 9)	**Y13**, N41, G71, E72, **K75**, R94, **G102**, **G103**, **G104**, V105, **D108**, F112, S114, **R119**, G120, **P127**, **T128**, T129, L130, L131, Q133, V134, **D135**, S136, S137, **G139**, G140, **K141**, T142, N145, K150, **N151**, **G154**, F156, **P159**, V162, D165, L172, G180, **E183**, **K186**, **K225**, V229, **D232**, E235, **R239**, **L242**, **N243**, **G245**, **H246**, T247, **H250**, A251, **E253**, **H263**, **G264**, A266, V267, **G270**, **D320**, **K321**, **K322**
Conserved (Score = 8)	V6, I15, I17, V44, Y48, D70, C96, A100, G107, M109, G111, A113, A115, C116, F117, Q118, V121, F123, Q125, S132, V138, G143, I144, H146, G149, M152, L153, A155, Q158, L169, T171, E176, A179, A182, Y187, A188, L190, D192, F195, L199, V218, A226, I228, E233, A240, L244, F248, G249, I252, L262, E265, A268, T269, M271, A274, A275, S278, I296, L301, P302, M317, V331, G337, I354
Variable (Score = 1-3)	A208, R209, A223, E238, L314, Y316, S285, Q335, H5, E7, G9, F16, Q20, P23, K24, Q25, L26, E28, P29, I31, H32, G33, Q34, P46, S50, H51, Q53, E54, A55, E57, S58, L59, K61, T62, A64, T65, Q78, L82, A90, Y122, A167, N170, E174, E193, D194, V197, E201, M203, D204, G205, V207, D210, A211, D212, L213, A215, Y219, R227, R280, N286, E287, A290, K293, K294, Q297, S304, Q307, P309, L310, D311, D312, G315, A318, N325, Q327, K334, Q338, V340, K343, D346, V347, E348, L349, K351, Q352, L355, A356, N357, Q358, H359, G360

Table S7. Amino acids of DHQ and NAD binding sites of DHQS from *A. baumannii* (PDB: 5EKS), with their ConSurf scores and maximum identity.

DHQ binding site			NADPH binding site					
Residue	Score	Max identity (%)	Residue	Score	Max identity (%)	Residue	Score	Max identity (%)
D135	9	100	N41	9	46.4	T128	9	100
K141	9	100	T43	5	49	T129	9	84.2
N151	9	100	V44	8	51.5	L131	9	91.3
K225	9	100	L47	5	62.2	D135	9	100
R239	9	100	Y48	8	60.7	S136	9	68.4
			D70	8	68.9	K141	9	100
			E72	9	98.5	N151	9	100
			K75	9	100	Q168	5	27.0
			G103	9	100	T171	8	88.3
			G104	9	100	L172	9	96.9
			V105	9	72.4	P173	7	82.1
			D108	9	100	E176	8	90.8

2.3.2. *Heatmaps and binding sites detected by FTMap and Sitemap*

Table S8. Amino acid residues and the corresponding clusters of the DHQS from *A .baumannii* (PDB: 5EKS) identified by FTMap and SiteMap. Conserved and highly conserved residues are in bold (scores 8 and 9).

Site	Residues	Site	Residues
F1	**G103**, **G104**, **V105**, **T129**, L130, L131, S132, **E176**, A179, **G180**, L262, **H263**, **G264**	S1	**N41**, T43, **V44**, L47, **Y48**, **D70**, **G71**, **E72**, K73, **K75**, D76, I77 , Q78, **G102, G103**, **G104**, **V105**, I106, G107, **D108**, F123, **Q125**, **T128**, **T129**, **L130**, **L131**, **S132**, **D135**, **S136**, **V138**, **G139**, **G140**,

Site	Residues	Site	Residues
			**K141**, **T142**, **G143**, **N145**, **H146**, P147, **G149**, **K150**, **N151**, **G154**, **A155**, **F156**, Q157, **P159**, Q168, **L169, T171**, **L172**, P173, **E176**, **A179**, **K186**, **Y187**, **K225**, **R239**, **A240**, **L242**, **N243**, **H246**, **T247**, **H250**, **E253**, S254, G257, Y258, G259, W261, **L262**, **H263**, **G264**, **D320**, **K322**
F2	L131, **D135**, **E183**, **K186**, Y187, **K225**, **L242**, **N243**, **H246**, H263	S2	A240, **N243**, **G245**, **H246**, **T247**, **D320**, **K321**, **K322**, V323, G326, Q327, L328, R329, L330, V331, I341
F3	**D108**, Q125, D135, S136, S137, V138, **G139**, **G140**, **K141**, K225		
F4	N41, **D70**, G71, **E72**, **G102**, G103, G104, V105, I106, T129, E176		
F5	G104, D108, L131, D135, S136, S137, V138, K141, K225		
F6	E72, **K75**, G104, V105, D108, L131, S136, **N151**		
F7	K75, D108, K141, **T142**, G143, N151		
F9	**E253**, Y258, G259, W261, L262, H263		
F10	K141, K225, **R239**, L242		
F8	L47, Y48, T128, Q168, T171, L172		

2.4. DHQase

2.4.1. Length analysis

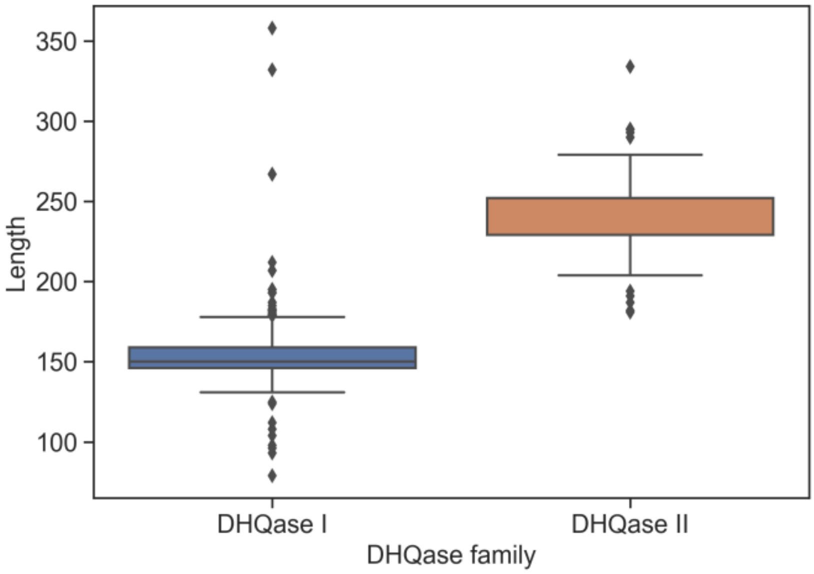

Figure S4. Comparison of protein lengths between type I and II DHQase counted in the number of amino acids that constitute each protein.

2.4.2. Type I

- Conservation analysis by ConSurf

Table S9. Highly conserved residues (conservation score 9), conserved residues (conservation score 8) and variable residues (conservation scores 1–3) of the DHQase I from *S. enterica* (PDB: 4CNN) identified by the ConSurf server [37]. Residues in bold print show no variation.

Residue classification	Residues
Highly conserved (Score = 9)	D43, **E46**, **R48**, **D50**, T80, **R82**, E86, G87, **G88**, **D114**, E116, S141, **H143**, D144, F145, T148, **P149**, M161, **K170**, A172, **D180**, V181, L184, T188, **M205**, **G209**, **R213**, **G220**, S221, A233, **G235**, **Q236**
Conserved (Score = 8)	I18, V20, S21, P76, S83, Y97, D111, M154, V173, M174, P175, L185, A187, T202, M203, S204, S212, T224, F225, S232, P234, L242
Variable (Score = 1-3)	L9, I10, R25, K31, E33, Y37, R38, D54, I55, S57, Q59, S60, V67, D70, D74, Q90, I92, T94, Q95, H96, L98, R102, A103, A104, I105, S107, L109, V110, G120, K125, A126, H134, N135, H146, A151, L156, A163, S177, H179, T186, A196, D197, E208, A223, Q230, D241, S244, M247, I248, N251, A252, T3, T99, D106, K2, T5, L35, A40, T41, L62, T63, A71, I75, T91, D123, D129, A133, S150, H194, M15, I27, A32, A36, E39, M53, A56, T58, R66, P73, A84, K85, A122, Y130, N240

Table S10. Amino acids of the DHQase I binding sites from *S. enterica* (PDB: 4CNN) identified by the ConSurf server [37] with their ConSurf scores and maximum identity.

DHQ binding site		
**Residues**	**Score**	**Max identity (%)**
S21	8	55.7
E46	9	100
R48	9	100
T80	9	98.4
R82	9	100
H143	9	100
K170	9	100
M203	8	59.7
R213	9	100
F225	8	83.9

- Heatmaps and binding sites detected by FTMap and Sitemap

Table S11. Amino acid residues and the corresponding clusters of DHQase I from *S. enterica* (PDB: 4CNN) identified by FTMap and SiteMap. Conserved and highly conserved residues are shown in bold (scores 8 and 9).

Site	Residues	Site	Residues
F1	I19, **V20**, **S21**, M23, **E46**, **R48**, **R82**, **K170**, **R213**, **F225**, G226, A227, **Q236**	S1	**V20, S21**, M23, Y37, **E46**, **R48**, **T80**, **R82**, **E86**, **G87**, **G88**, E89, **H143**, **F145**, **K170**, **A172**, I201, **M203**, **M205**, A206, **R213**, **F225**, G226, A227, V228, **G235**, **Q236**
F2	**R48**, **R82**, **E86**, **G87**, **G88**, **H143**, **D144**, **F145**, **A172**, **M205**	S2	K2, G12, E13, G14, M15, P16, K17, Q192, A196, R198, P199, V200, G216, E217, **G220**, **S221**, A222
F3	**M205**, A206, **R213**, **G235**, **Q236**	S3	S83, A84, K85, **E86**, T91, I92, T94, Y97, **E116**, F118, T119
F4	**S21**, **E46**, **R48**, **T80**, **R82**, **E86**, **G87**, **H143, K170, A172**, **M203**, **M205**,		

Site	Residues	Site	Residues
	**R213**, **F225**		
F5	**V20**, **S21**, L22, M23, Y37, G226, A227, V228, **Q236**		
F6	Y37, R38, A40, T41, F42, M72, P73, D74, I75		
F7	G12, E13, G14, M15, P16, K17, Q192, G216, E217, **G220**, **S221**, A222		
F8	K85, **E86**, **G87**, F118, N142, **H143**, **D144**, **F145**, H146		
F9	I11, G12, G12, G12, **S83**, I92, T94, **Y97**, **E116**, T119		

2.4.3. Type II

- Conservation analysis by ConSurf

Table S12. Highly conserved residues (conservation score 9), conserved residues (conservation score 8) and variable residues (conservation scores 1–3) of the DHQase II from *M. tuberculosis* (PDB: 4KIW), identified ConSurf server [37]. Residues in bold print show no variation.

Residue classification	Residues
Highly conserved (Score = 9)	N9, **G10**, P11, **N12**, G14, L16, **G17**, R19, **Y24**, **G25**, **Q51**, D53, **E55**, **N75**, H81, S83, A85, **D88**, A89, **E99**, **H101**, S103, N104, **R108**, E109, F111, **R112**, S115, **G127**, G129, Y133, A136
Conserved (Score = 8)	L13, E20, T28, S52, V72, L74, A76, G77, G78, T80, T82, V84, V100, S118, A121, I125, G132
Variable (Score = 1-3)	E31, A47, A56, P71, L79, A107, L3, I4, R18, A22, T27, L43, G44, L45, V49, R50, S54, Q57, W61, Q64, A66, D67, A69, E70, A91, E110, I120, T122, V126, I130, L134, L135, R138, Y139, L140, A141, T146, G26, D30, A34, E39, E42, K46, V48, S94, Q131, L35, E37, R38, A41, A68, E142, H143, V144, G145

Table S13. Amino acids of 3-dehydroquinate binding site of DHQase II from *M. tuberculosis* (PDB: 4KIW), with their ConSurf scores and maximum identity.

DHQase II binding site		
**Residues**	**Score**	**Max identity (%)**
P11	9	98.8
L13	8	92.5
R19	9	99,4
N75	9	100
G77	8	61,5
G78	8	75,2
H81	9	98,1
H101	9	100
I102	7	53,4
S103	9	98,8
V105	6	62,1
R108	9	100
R112	9	100

- Heatmaps and binding sites detected by FTMap and Sitemap

Table S14. Amino acid residues and the corresponding clusters of DHQase II from *M. tuberculosis* (PDB: 4KIW), identified by FTMap and SiteMap. Conserved and highly conserved residues are in bold (scores 8 and 9).

Site	Residues	Site	Residues
F1	**P11**, **N12**, **L13**, G14, R15, **L16**, **G17**, R18, **N75**, **G77**, **G78**	S1	**P11**, **N12**, **L13**, R15, **L16**, **G17**, R18, **N75**, **G77**, **G78**, **H81**, **H101**, I102, **S103**, V105, **R108**, **R112**
F2	**L13**, **N75**, **G77**, **H101**, I102, **S103**, V105, **R108**		
F3	**N75**, **G77**, **G78**, **T80**, **H81**, **H101**, V105, **R112**		
F4	**P11**, **N12**, **L13**, **N75**, **G77**, **G78**		
F5	I98, T122, G123, V124, **I125**, **A136**, Y139		
F6	**G14**, **I125**, L135, **A136**, R138, Y139		
F7	C90, A91, L93, S94, A95, P96, L97, I120, **A121**, T122		
F8	H81, **E99**, H101, V105, H106, R112, R113, H114, **S115**, **S118**, V124, V126		
F9	H106, V124, I125, V126, **G127**, L128		
F10	I102, S103, **N104**, G127, L128, **G129**		
F11	H29, V33, V49, R50, **Q51**		

2.5. SDH

2.5.1. Conservation analysis by ConSurf

Table S15. Highly conserved residues (conservation score 9), conserved residues (conservation score 8) and variable residues (conservation scores 1–3) of the SDH from *M. tuberculosis* (PDB: 4P4G), identified by the ConSurf server [37]. Residues in bold print show no variation.

Residue classification	Residues
Highly conserved (Score = 9)	V10, G12, P14, H17, S18, S20, P21, H24, Y38, G61, S63, T65, P67, K69, A83, A89, N90, T91, N103, T104, D105, G108, G124, G126, G127, T128, S190, I192, D212, Y215, T220, **G236**, M239, Q243, A244
Conserved (Score = 8)	G9, I15, L23, L25, A27, Y28, V64, M66, A72, A76, V86, G87, S88, L92, A101, D102, I106, V109, A112, L113, S125, A129, A131, A132, G135, L136, A148, R149, N150, A154, V189, T191, P193, V196, A213, I214, L222, A223, V226, S235, L240, L241, F245, Q247, F251, T252, L265
Variable (Score = 1-3)	A46, A71, G165, P255, A264, Q22, R29, T37, G54, F75, V166, T168, A179, A198, G227, H242, D269, H34, G44, A45, E47, P49, V50, G53, P57, R74, D84, L85, P96, G111, G114, A115, A116, A117, G118, H119, T142, D143, S151, D152, A155, R156, D159, L160, T162, R163, V164, R169, F170, A172, F173, D174, S175, L178, D180, A181, V182, A183, A184, A197, G199, Y200, G202, T203, L204, A205, I207, W218, A224, S228, A229, R232, L254, R258, A260, C263, A266, A267, L268, A167, C171, G176, G177, A206

Table S16. Amino acids of the shikimate and NADPH binding sites of SDH from *M. tuberculosis* (PDB: 4P4G), with their ConSurf scores and maximum identity.

**Shikimate binding site**			**NADPH binding site**		
Residues	Score	Max identity (%)	Residues	Score	Max identity (%)
V10	9	70.6	M66	8	40
S18	9	92.6	G124	9	99
S20	9	92.6	S125	8	67
S63	9	72.5	G126	9	100
V64	8	73.0	G127	9	97
T65	9	79.9	T128	9	65
K69	9	99.0	A148	8	58
N90	9	99.0	R149	8	87
D105	9	99.5	F173	1	37
Y215	9	91.2	S190	9	75
L240	8	79.4	T191	8	47
Q243	9	98.0	I192	9	78
Q247	8	47.5	P193	8	38
			A194	6	29
			V196	8	40
			A197	1	19
			Y200	1	15
			D212	9	96
			A213	8	29
			I214	8	35
			Y215	9	91
			G236	9	100
			M239	9	93
			L240	8	79

2.5.2. Heatmaps and binding sites detected by FTMap and Sitemap

Table S17. Amino acid residues and the corresponding clusters of SDH from *M. tuberculosis* (PDB: 4P4G), identified by FTMap and SiteMap. Conserved and highly conserved residues are in bold (score 8 and 9).

Site	Residues	Site	Residues
F1	**V10**, **H17**, **S18,** R19, **S20**, **S63**, V64, T65, K69, N90, D105, **Y215**, L240, Q243, **Q247**	S1	**V10**, **P14**, **I15**, **H17**, **S18**, **S20**, **H24**, **Y38**, **S63**, **V64**, **T65**, **M66**, **P67**, **K69**, F70, **N90**, **D105**, **G124**, **S125**, **G126**, **G127**, **T128**, **A129**, **A148**, **R149**, **N150**, F173, **T191**, **I192**, **P193**, A194, E195, **A213**, **I214**, **Y215**, D216, P217, **G236**, **M239**, **L240**, **Q243**, **A244**, **Q247**
F2	**H17**, A213, **I214**, Y215, D216	S2	F70, L85, **V86**, **G87**, **S125**, P130, D152, K153, R156, L157, L160, R163
F3	**V64**, **T65**, **K69**, **N90**, **D105**, **T128**, **A213**, **G236**, **L240**, **Q243**	S3	R19, **Y215**, D216, P217, W218, L237, D269
F4	**T65**, **K69**, **G126**, **G127**, **T128**, **A129**, **T191**, **I192**, A213, I214		
F5	**G124**, S125, **G126**, **A148**, **R149**, **N150**, T191, I192		
F6	D105, A213, Y215, G236, L240, Q243		
F7	S13, **P14**, **H17**, T65		

Site	Residues	Site	Residues
F8	**P67**, G68, K69, F70, G127		
F9	F70, L85, **V86**, **G87**, **S125**, **G126**, P130, K153, R156, L157, L160, R163		
F10	V60, **G61**, V93, R94, T95, Q250, **F251**		

2.6. SK

2.6.1. Analysis of length

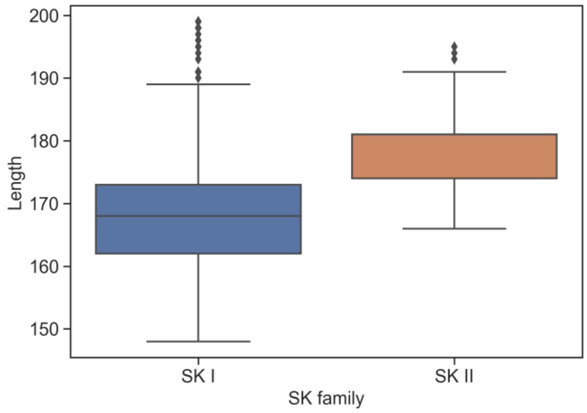

Figure S5. Comparison of protein lengths between type I and II SK counted in the number of amino acids that constitute each protein.

2.6.2. Conservation analysis by ConSurf

Table S18. Highly conserved residues (conservation score 9), conserved residues (conservation score 8) and variable residues (conservation scores 1–3) of SK II from *M. tuberculosis* (PDB: 2IYQ), identified by ConSurf server [37]. Residues in bold show no variation.

Residue classification	Residues
Highly conserved (Score = 9)	**G9**, P11, G12, G14, **K15**, T17, D32, **D34**, G53, E54, F57, R58, E61, L78, G79, G80, G81, L100, R110, R117, P118, R136, Y140
Conserved (Score = 8)	A5, L7, S13, S16, G19, L22, A23, T33, I37, E38, I45, I48, F49, V65, V75, S77, A82, V88, R89, L92, V97, T111, L119, L120, L132, A144, T150, I162
Variable (Score = 1-3)	T96, P123, G94, G27, G29, L31, V35, R40, T51, Q55, R59, A70, D71, P86, A90, A91, A131, A134, M146, R152, L163, S164, R165, Q167, V168, P169, S170, P171, S172, A174, A175, E173, T176

Table S19. Amino acids of the shikimate and ATP binding site of the SK II from *M. tuberculosis* (PDB: 2IYQ) with their ConSurf scores and maximum identity.

**Shikimate binding site**			**ATP binding site**		
**Residues**	**Score**	**Max identity (%)**	**Residues**	**Score**	**Max identity (%)**
P11	9	58.1	L10	6	37.8
D34	9	100	P11	9	58.1
I45	8	60.3	G12	9	98.3
F57	9	97.1	S13	8	54.4
R58	9	96.7	G14	9	99.6
G79	9	96.7	K15	9	100
G80	9	96.3	S16	8	49
G81	9	97.9	T17	9	92.1
P118	9	97.1	R110	9	97.1
L119	8	71.9	R117	9	97.1
R136	9	98.3	R153	5	20.2
			N154	5	36.9
			P155	5	47.1

2.6.3. Heatmaps and binding sites detected by FTMap and Sitemap

Table S20. Amino acid residues and the corresponding clusters of SK from *M. tuberculosis* (PDB: 2IYQ) identified by FTMap and SiteMap. Conserved and highly conserved residues are in bold (score 8 and 9).

**Site**	**Residues**	**Site**	**Residues**
F1	**P11**, **K15**, **D34**, **I45**, **F49**, **F57**, **R58**, **G79**, **G80**, **G81**, V116, **R117**, **P118**, **L119**, **L120**, **L132**, **R136**	S1	L10, **P11**, **G12**, **S13**, **G14**, **K15**, **S16**, **T17**, I18, **D32**, **D34**, **E38**, S44, **I45**, A46, **F49**, **F57**, **R58**, **L78**, **G79**, **G80**, **G81**, **R110**, V116, **R117**, **P118**, **L119**, **L120**, **L132**, **R136**, **T150**, R153, N154, P155, V158
F2	K15, **S16**, **D32**, D34, V35, **S77**, **L78**, G79, R117		
F3	L10, **G12**, **S13**, **G14**, K15, S16, **T17**, R117		
F4	S13, G14, T17, I18, **R110**, **T150**, R153, N154, P155, V158		
F5	S16, D32, D34, V35, **E38**, I45, A46, V116		
F6	D34, I45, F57, R58, **E61**, G79, G80, G81		
F7	G12, G14, T17, R110		
F8	L10, Y99, **L100**, E101, I102, M133, A137, **Y140**		
F9	L31, A36, R40, V64, A67, A68		
F10	L30, L31, D32, **T33**, V35, A36, Q39		

2.6.4. Conservation analysis by ConSurf

Table S21. Highly conserved residues (conservation score 9), conserved residues (conservation score 8) and variable residues (conservation scores 1–3) of SK II from *M. tuberculosis* (PDB: 2IYQ), identified by ConSurf server [37]. Residues in bold show no variation.

Residue classification	Residues
Highly conserved (Score = 9)	**G9**, P11, G12, G14, **K15**, T17, D32, **D34**, G53, E54, F57, R58, E61, L78, G79, G80, G81, L100, R110, R117, P118, R136, Y140
Conserved (Score = 8)	A5, L7, S13, S16, G19, L22, A23, T33, I37, E38, I45, I48, F49, V65, V75, S77, A82, V88, R89, L92, V97, T111, L119, L120, L132, A144, T150, I162
Variable (Score = 1-3)	T96, P123, G94, G27, G29, L31, V35, R40, T51, Q55, R59, A70, D71, P86, A90, A91, A131, A134, M146, R152, L163, S164, R165, Q167, V168, P169, S170, P171, S172, A174, A175, E173, T176

Table S22. Amino acids of the shikimate and ATP binding site of the SK II from *M. tuberculosis* (PDB: 2IYQ) with their ConSurf scores and maximum identity.

Shikimate binding site			ATP binding site		
Residues	Score	Max identity (%)	Residues	Score	Max identity (%)
P11	9	58.1	L10	6	37.8
D34	9	100	P11	9	58.1
I45	8	60.3	G12	9	98.3
F57	9	97.1	S13	8	54.4
R58	9	96.7	G14	9	99.6
G79	9	96.7	K15	9	100
G80	9	96.3	S16	8	49
G81	9	97.9	T17	9	92.1
P118	9	97.1	R110	9	97.1
L119	8	71.9	R117	9	97.1
R136	9	98.3	R153	5	20.2
			N154	5	36.9
			P155	5	47.1

2.6.5. Heatmaps and binding sites detected by FTMap and Sitemap

Table S23. Amino acid residues and the corresponding clusters of SK from *M. tuberculosis* (PDB: 2IYQ) identified by FTMaps and SiteMaps. Conserved and highly conserved residues are in bold (score 8 and 9).

Site	Residues	Site	Residues
F1	**P11**, **K15**, **D34**, **I45**, **F49**, **F57**, **R58**, **G79**, **G80**, **G81**, V116, **R117**, **P118**, **L119**, **L120**, **L132**, **R136**	S1	L10, **P11**, **G12**, **S13**, **G14**, **K15**, **S16**, **T17**, I18, **D32**, **D34**, **E38**, S44, **I45**, A46, **F49**, **F57**, **R58**, **L78**, **G79**, **G80**, **G81**, **R110**, V116, **R117**, **P118**, **L119**, **L120**, **L132**, **R136**, **T150**, R153, N154, P155, V158
F2	K15, **S16**, **D32**, D34, V35, **S77**, **L78**, G79, R117		
F3	L10, **G12**, **S13**, **G14**, K15, S16, **T17**, R117		
F4	S13, G14, T17, I18, **R110**, **T150**, R153, N154, P155, V158		
F5	S16, D32, D34, V35, **E38**, I45, A46, V116		
F6	D34, I45, F57, R58, **E61**, G79, G80, G81		
F7	G12, G14, T17, R110		
F8	L10, Y99, **L100**, E101, I102, M133, A137, **Y140**		
F9	L31, A36, R40, V64, A67, A68		
F10	L30, L31, D32, **T33**, V35, A36, Q39		

2.7. *EPSPS*

2.7.1. Conservation analysis by ConSurf

Table S24. Highly conserved residues (conservation score 9), conserved residues (conservation score 8), and variable residues (conservation scores1–3) of the EPSPS from *E. coli* (PDB: 2AA9) identified by the ConSurf server [37]. Residues in bold print show no variation.

Residue classification	Residues
**Highly conserved (Score = 9)**	S21, K22, S23, N26, **R27**, D49, G61, N94, A95, G96, T97, R100, M121, **R124**, P125, L133, P150, S169, S170, Q171, F172, T174, S197, Y200, T204, E240, D242, S244, S245, A246, S269, D273, D313, M316, N336, R338, K340, E341, R344, L352, G356, D364, **D384**, H385, R386, A388, M389, T412, P414
**Conserved (Score = 8)**	P19, V24, S25, L29, A33, L45, S47, V50, M53, A56, L57, L60, G93, A98, P101, A104, L106, G117, E118, R120, I126, A132, G137, A138, L173, A175, L176, L177, M178, P199, I201, I203, M208, G212, G241, A243, S247, A252, I265, Q271, G272, M283, G284, P312, A314, I318, A319, T320, A322, A325, T329, L331, N333, I334, W337, V339, T342, D343, L345, A347, M348, E351, I368, P370, T381, M387, S392, A395, L396, I403, T409, K411, F413, Y416
**Variable (Score = 1-3)**	N111, D131, R134, K261, R275, D291, E360, L374, P400, M1, E2, S3, T5, Q7, P8, I9, A10, R11, D13, N17, H36, K38, V40, T42, N55, T58, A59, S63, T65, L66, S67, A68, D69, R70, T71, C73, E74, I76, N78, G80, P81, L82, H83, A84, E85, G86, A87, L88, E89, G109, S110, D112, V114, P119, G127, L135,

Residue classification	Residues
	K139, T141, L143, Q145, E146, Y148, R152, Q154, G156, T158, N161, D163, P184, E185, D186, V188, R190, K192, D194, N206, K209, T210, E214, E216, N217, Q218, H219, Y220, Q221, Q222, V224, K226, G227, G228, S230, Y231, S233, G235, T236, L238, T259, R267, D278, E281, K282, T286, C288, W289, G290, Y293, S295, T297, R298, G299, E300, N302, K326, T328, T330, R332, E358, H363, R367, T369, P371, E372, K373, N375, F376, E378, A380, T399, D415, E418, A421, S424, Q425, A427, R72, R422, A426

Table S25. Amino acids of shikimate and PEP binding sites of EPSPS from *E. coli* (PDB: 2AA9) with their ConSurf [37] scores and maximum identity.

Shikimate binding site			PEP binding site		
Residues	Score	Max identity (%)	Residues	Score	Max identity (%)
K22	9	99.5	K22	9	99.5
S23	9	98.5	N94	9	70.9
R27	9	100	A95	9	63.1
T97	9	94.2	G96	9	98.5
S170	9	99,5	T97	9	94.2
Q171	9	66.0	R124	9	100
Y200	9	56.1	Q171	9	66.0
D313	9	76.6	D313	9	76.6
K340	9	75.7	E341	9	99.0
			R344	9	99.0
			H385	9	99.5
			R386	9	99.5
			K411	8	64.6

2.7.2. Heatmaps and binding sites detected by FTMap and Sitemap

Table S26. Amino acid residues and the corresponding clusters of EPSPS from *E. coli* (PDB: 2AA9) identified by FTMap and SiteMap. Conserved and highly conserved residues are in bold (score 8 and 9).

Site	Residues	Site	Residues
F1	N17, **P19**, G20, **L45**, D46, D69, T71, **F413**, **P414**, D415, Q419	S1	**K22**, **R27**, **D49**, **N94**, **A95**, **G96**, **T97**, **R100**, **R124**, **P125**, V168, **S169**, **S170**, **Q171**, V196, **S197**, **Y200**, **P312**, **D313**, **N336**, **V339**, **K340**, **E341**, **R344**, **H385**, **R386**, **K411**
F2	L18, **P19**, G20, N43, L44, **L45**, D46, T71	S2	D48, **D49**, H52, **G93**, **N94**, **A95**, **E118**, P119, **R120**, **E341**, **T342**, Y382, N383, **D384**, **H385**, D405, C408, **K411**
F3	**S21**, **K22**, **S23**, **V24**, **Y200**, **D242**, **A243**, **S244**, **Q271**, **D273**	S3	N17, L18, N43, L238, V239, **E240**, T263, G264, **I265**, G266, N268, **S269**, D291, D292
F4	V168, **S169**, **S170**, **Q171**, V196, **S197**, Y200, **P312**, **N336**, **K340**	S4	N17, **P19**, G20, N43, L44, **L45**, D46, T71, **F413**, **P414**, D415, Q419
F5	**K22**, **S23**, **R27**, **T97**, **Q171**, **Y200**, **D313**, K340	S5	K256, L323, F324, P371, E372, K373, L374, S397, D398, T399
F6	**K22**, **D49**, **N94**, **A95**, **G96**, T97, **R124**, **Q171**, **E341**, **K411**		
F7	**K22**, D48, **D49**, H52, **G93**, **N94**, **A95**,		

Site	Residues	Site	Residues
	**G96**, **T97**, **E118**, **R124**, **Q171**, **E341**, N383, **D384**, **H385**, C408, A410, **K411**		
F8	L323, F324, **A325**, **P370**, P371, E372, K373, L374		
F9	**P199**, D202, **S269**, Q271, **G272**, I274, R275		
F10	L238, **E240**, **G241**, G264, **I265**, G266, N268, S269, D291, D292		
F11	N206, L207, T210, Y237, L238, V239, E240		
F12	**R120**, E123, **P125**, **I126**, G127, H128, L129, N147, Y148, V168, **F172**, **V339**		
F13	**R120**, E123, **P125**, Y148, V339		

2.8. CS

2.8.1. Conservation analysis by ConSurf

Table S27. Highly conserved residues (conservation score 9), conserved residues (conservation score 8), and variable residues (conservation scores 1–3) of CS from *M. tuberculosis* (PDB: 2QHF) identified by the ConSurf server [37]. Residues in bold show no variation.

Residue classification	Residues
Highly conserved (Score = 9)	T6, **G8**, E9, **S10**, **H11**, G12, E20, R39, **R40**, G43, G45, R49, **E53**, **D55**, **G62**, T68, G70, **P72**, N79, **W85**, M89, **R112**, **P113**, **H115**, A116, **D117**, G120, K123, D128, R130, L133, E134, R135, **S137**, A138, **R139**, E140, T141, R144, V145, A146, A151, L155, T218, **G220**, G221, G233, **G235**, R243, L244, D245, A249, M253, Q256, A257, I258, **K259**, V261, E262, **G264**, G266, G274, S275, H278, D279, R290, N293, **G296**, **G297**, E299, G300, **G301**, M302, T303, G305, **K315**, P316, I317, T319, V320, L324, Q340, **R341**, S342, **D343**, A346, A349, A350, V353, E355, A359
Conserved (Score = 8)	L2, W4, I5, A7, A14, L15, V16, G21, V23, A24, G25, L37, R41, Y44, R46, M50, T51, F52, V57, V59, S61, R64, H65, G66, L69, G71, I73, I77, E81, W82, K84, R104, P111, G114, P131, V132, A136, A142, A143, A147, G148, T149, R152, V167, G171, S192, V194, R195, D198, M205, I209, K213, D217, E224, P231, S236, F237, S239, S246, L248, A251, G254, I255, G260, I263, F267, A277, G287, T292, A295, N304, Q306, A313, M314, S318, T326, I338, V344, C345, V347, P348, G351, V352, V354, M357, V361, A363
Variable (Score = 1-3)	A325, V389, L186, D215, R13, H27, D35, S67, T87, A91, P96, A97, R156, E162, A172, A174, Y176, E177, G178, P179, P181, R182, A183, E184, P187, A188, A191, K199, A200, A203, D204, A207, A211, L228, Q268, R271, Y282, P283, G284, P285, D286, V289, S291, P307, A330, L375, A376, Q379, R380, I382, A383, A384, Q386, R387, S388, A390, D391, E393, A394, P395, A396, A397, R398, G401, P180, V399, S400

Table S28. Amino acids of EPSP binding site of CS from *M. tuberculosis* (PDB: 2QHF) active and binding sites with their ConSurf [37] scores and maximum identity.

EPSP binding site			FMN binding site		
**Residues**	**Score**	**Max identitity (%)**	**Residues**	**Score**	**Max identitity (%)**
H11	9	100	R46	8	81,6
R40	9	100	R49	9	57
R46	8	81,6	G114	8	90,8
R49	9	57	H115	9	100
R112	9	100	A116	9	97,6
S137	9	100	A136	8	68,6
A138	9	95,7	A138	9	95,7
R139	9	100	I255	8	87,9
I317	9	55,1	Q256	9	70
Q340	9	58,5	A257	9	95,2
R341	9	100	M314	8	41,1
			K315	9	100
			I317	9	55,1
			S318	8	64,7
			T319	9	56,5
			R341	9	100
			S342	9	58,5
			D343	9	100

2.8.2. Heatmaps and binding site analysis

Table S29. Amino acid residues and the corresponding clusters of the CS from *M. tuberculosis* (PDB: 2QHF) identified by FTMap and SiteMap. Conserved and highly conserved residues are in bold (scores 8 and 9).

Site	Residues	Site	Residues
F1	N17, **P19**, G20, **L45**, D46, D69, T71, **F413**, **P414**, D415, Q419	S1	**K22**, **R27**, **D49**, **N94**, **A95**, **G96**, **T97**, **R100**, **R124**, **P125**, V168, **S169**, **S170**, **Q171**, V196, **S197**, **Y200**, **P312**, **D313**, **N336**, **V339**, **K340**, **E341**, **R344**, **H385**, **R386**, **K411**
F2	L18, **P19**, G20, N43, L44, **L45**, D46, T71	S2	D48, **D49**, H52, **G93**, **N94**, **A95**, **E118**, P119, **R120**, **E341**, **T342**, Y382, N383, **D384**, **H385**, D405, C408, **K411**
F3	**S21**, **K22**, **S23**, **V24**, **Y200**, **D242**, **A243**, **S244**, **Q271**, **D273**	S3	N17, L18, N43, L238, V239, **E240**, T263, G264, **I265**, G266, N268, **S269**, D291, D292
F4	V168, **S169**, **S170**, **Q171**, V196, **S197**, Y200, **P312**, **N336**, **K340**	S4	N17, **P19**, G20, N43, L44, **L45**, D46, T71, **F413**, **P414**, D415, Q419
F5	**K22**, **S23**, **R27**, **T97**, **Q171**, **Y200**, **D313**, K340	S5	K256, L323, F324, P371, E372, K373, L374, S397, D398, T399
F6	**K22**, **D49**, **N94**, **A95**, **G96**, T97, **R124**, **Q171**, **E341**, **K411**		
F7	**K22**, D48, **D49**, H52, **G93**, **N94**, **A95**, **G96**, **T97**, **E118**, **R124**, **Q171**, **E341**, N383, **D384**, **H385**, C408, A410, **K411**		
F8	L323, F324, **A325**, **P370**, P371, E372, K373, L374		

Site	Residues	Site	Residues
F9	**P199**, D202, **S269**, Q271, **G272**, I274, R275		
F10	L238, **E240**, **G241**, G264, **I265**, G266, N268, S269, D291, D292		
F11	N206, L207, T210, Y237, L238, V239, E240		
F12	**R120**, E123, **P125**, **I126**, G127, H128, L129, N147, Y148, V168, **F172**, **V339**		
F13	**R120**, E123, **P125**, Y148, V339		

2.9. Druggability analyses

2.9.1. Data

Table S30. Druggabilities and important physicochemical characteristics of the detected binding sites.

Enzyme	PDB ID	Site	Dscore	SiteScore	Volume	Exposure	Enclosure	Contact	Phobic	Philic	Balance	Don/Acc	SBS
DAHPS	1GG1	1	0.799	0.873	176.0	0.465	0.799	1.012	0.975	1.137	0.857	3.144	No
DAHPS	1GG1	2	0.599	0.921	138.7	0.400	0.849	1.142	0.151	1.902	0.08	0.364	Yes
DAHPS	1GG1	3	0.803	0.811	133.9	0.608	0.711	0.848	1.701	0.668	2.545	0.254	No
DAHPS	1GG1	4	0.520	0.640	106.9	0.716	0.627	0.925	0.383	1.215	0.315	2.856	No
DAHPS	1GG1	5	0.456	0.599	69.6	0.603	0.669	0.996	0.426	1.218	0.35	2.041	No
DAHPS	1GG1	6	0.839	0.906	46.3	0.431	0.998	1.485	4.135	0.815	5.074	1.17	No
DAHPS	1RZM	1	0.770	1.058	282.2	0.43	0.784	1.123	0.19	1.964	0.097	0.372	Yes
DAHPS	1RZM	2	0.662	0.703	88.8	0.653	0.709	0.922	1.609	0.718	2.242	2.613	No
DAHPS	4UC5	1	0.915	1.026	332.5	0.556	0.737	0.943	0.425	1.435	0.296	0.599	Yes
DAHPS	4UC5	2	1.005	0.982	205.8	0.558	0.693	0.933	1.341	0.943	1.422	0.847	No
DAHPS	4UC5	3	0.621	0.665	75.8	0.617	0.584	0.837	0.594	1.017	0.584	0.416	No
DAHPS	4UC5	4	0.594	0.637	85.7	0.708	0.582	0.794	0.555	0.906	0.613	0.632	No
DAHPS	4UC5	5	0.624	0.649	90.1	0.746	0.550	0.631	0.599	0.827	0.724	0.656	No
DAHPS	5E40	1	0.943	1.030	569.1	0.602	0.743	0.936	0.411	1.358	0.303	0.58	Yes
DAHPS	5E40	2	1.091	1.042	474.4	0.592	0.696	0.929	1.515	0.818	1.853	1.121	No
DAHPS	5E40	3	0.923	0.989	364.6	0.574	0.682	0.97	0.388	1.305	0.297	1.464	No
DAHPS	5E40	4	0.940	0.962	300.9	0.591	0.641	0.83	0.385	1.18	0.326	1.073	No
DAHPS	5E40	5	0.931	0.890	203.4	0.604	0.624	0.8	1.393	0.618	2.256	0.869	No
DAHPS	5E40	6	0.516	0.640	65.6	0.654	0.598	0.823	0.028	1.25	0.023	0.719	No
DAHPS	5E40	7	0.717	0.908	66.0	0.410	0.999	1.346	1.978	1.367	1.447	1.026	No
DAHPS	5E40	8	0.537	0.627	96.0	0.656	0.626	0.899	0.369	1.109	0.333	0.457	No
DAHPS	5E40	9	0.664	0.672	100.6	0.795	0.601	0.656	0.847	0.541	1.566	1.053	No
DAHPS	5HUD	1	0.894	1.049	285.6	0.510	0.772	1.006	0.358	1.56	0.23	0.523	Yes
DAHPS	5HUD	2	0.932	0.909	262.5	0.711	0.616	0.767	0.464	0.95	0.489	1.456	No
DAHPS	5HUD	3	1.001	0.970	194.9	0.556	0.736	0.959	2.028	0.663	3.057	0.905	No
DAHPS	5HUD	4	0.901	0.874	183.9	0.702	0.633	0.796	1.066	0.709	1.505	1.901	No
DAHPS	5HUD	5	0.760	0.866	196.2	0.668	0.67	0.913	0.356	1.332	0.267	0.497	No
DAHPS	5HUD	6	0.898	0.859	129.0	0.649	0.692	0.824	2.575	0.319	8.07	0.472	No
DAHPS	5HUD	7	0.639	0.691	144.6	0.729	0.673	0.891	0.819	0.909	0.902	1.216	No
DAHPS	5HUD	8	0.639	0.682	141.8	0.737	0.633	0.796	0.678	0.909	0.746	1.222	No

Enzyme	PDB ID	Site	Dscore	SiteScore	Volume	Exposure	Enclosure	Contact	Phobic	Philic	Balance	Don/Acc	SBS
DAHPS	5HUD	9	0.649	0.841	60.2	0.526	0.987	1.559	2.305	1.325	1.740	0.655	No
DAHPS	5UXM	1	0.949	1.016	519.6	0.534	0.722	0.945	0.414	1.300	0.318	0.463	Yes
DAHPS	5UXM	2	0.725	0.744	98.0	0.689	0.635	0.895	0.967	0.813	1.189	1.484	No
DAHPS	5UXM	3	0.563	0.707	119.5	0.683	0.671	0.858	0.053	1.308	0.041	0.789	No
DAHPS	5UXM	4	0.488	0.636	69.2	0.603	0.640	0.936	0.512	1.285	0.398	0.415	No
DAHPS	5UXM	5	0.528	0.581	102.0	0.812	0.55	0.704	0.356	0.946	0.376	6.093	No
DAHPS	5UXM	6	0.739	0.762	110.0	0.613	0.719	0.986	1.967	0.661	2.977	0.902	No
DAHPS	5UXM	7	0.539	0.639	85.1	0.659	0.654	0.819	0.474	1.129	0.42	0.456	No
DAHPS	5UXM	8	0.503	0.599	109.1	0.735	0.635	0.759	0.335	1.097	0.305	1.253	No
DAHPS	5UXM	9	0.546	0.849	56.5	0.364	0.989	1.353	1.361	1.672	0.814	0.81	No
DAHPS	5UXM	10	0.533	0.567	74.0	0.803	0.523	0.609	0.285	0.741	0.385	3.203	No
DHQS	1UJN	1	1.014	1.047	1163.6	0.544	0.769	1.014	0.664	1.188	0.559	0.853	Yes
DHQS	1UJN	2	0.766	0.812	103.7	0.471	0.799	1.135	1.914	0.875	2.187	1.083	No
DHQS	1UJN	3	0.689	0.698	72.8	0.611	0.579	0.807	0.907	0.706	1.285	1.135	No
DHQS	1UJN	4	0.535	0.686	122.7	0.659	0.725	0.989	0.37	1.273	0.29	1.023	No
DHQS	1UJN	5	0.448	0.616	64.2	0.611	0.640	0.957	0.51	1.33	0.383	0.535	No
DHQS	1UJN	6	0.503	0.561	76.1	0.725	0.538	0.728	0.411	0.973	0.422	1.588	No
DHQS	1XAG	1	0.971	1.068	880.2	0.395	0.800	1.06	0.802	1.376	0.583	0.637	Yes
DHQS	1XAG	2	1.062	0.972	205.0	0.683	0.594	0.770	1.912	0.4	4.777	2.054	No
DHQS	1XAG	3	0.526	0.585	101.1	0.832	0.604	0.793	0.492	0.85	0.579	1.408	No
DHQS	1XAG	4	0.605	0.626	111.3	0.833	0.576	0.739	1.037	0.618	1.676	0.584	No
DHQS	1XAG	5	0.48	0.565	49.3	0.638	0.635	0.979	0.683	1.019	0.670	1.341	No
DHQS	1XAG	6	0.556	0.617	49.3	0.632	0.658	0.913	1.488	0.828	1.798	0.599	No
DHQS	1XAH	1	0.993	1.056	839.4	0.459	0.781	1.015	0.775	1.275	0.608	0.74	Yes
DHQS	1XAH	2	1.209	1.135	248.1	0.433	0.792	1.062	2.910	0.529	5.498	2.007	No
DHQS	1XAH	3	0.800	0.806	110.3	0.652	0.676	0.864	1.294	0.748	1.732	9.397	No
DHQS	1XAH	4	0.563	0.602	59.9	0.727	0.543	0.668	0.63	0.851	0.741	0.286	No
DHQS	3QBE	1	0.954	1.033	1321.9	0.525	0.747	0.963	0.350	1.334	0.263	0.787	Yes
DHQS	3QBE	2	0.476	0.541	89.2	0.825	0.558	0.74	0.218	0.92	0.237	1.858	No
DHQS	5EKS	1	0.980	1.009	1126.6	0.579	0.711	0.919	0.624	1.186	0.526	0.736	Yes
DHQS	5EKS	2	0.662	0.714	110.7	0.664	0.656	0.902	0.631	1.045	0.604	0.507	No
DHQS	5HVN	1	0.962	1.040	1252.7	0.463	0.758	0.988	0.634	1.329	0.477	0.605	Yes

Enzyme	PDB ID	Site	Dscore	SiteScore	Volume	Exposure	Enclosure	Contact	Phobic	Philic	Balance	Don/Acc	SBS
DHQS	5HVN	2	1.000	0.971	322.6	0.638	0.643	0.864	0.502	1.001	0.502	0.866	No
DHQS	5HVN	3	0.645	0.662	143.5	0.817	0.567	0.654	0.373	0.721	0.517	1.196	No
DHQS	5HVN	4	0.616	0.640	90.3	0.710	0.572	0.781	1.097	0.715	1.535	0.730	No
DHQS	5HVN	5	0.556	0.612	62.4	0.603	0.628	0.93	1.049	0.834	1.259	1.378	No
DHQS	5HVN	6	0.466	0.570	57.9	0.732	0.511	0.697	0.109	1.194	0.092	0.658	No
DHQS	5HVN	7	0.585	0.623	93.4	0.803	0.581	0.716	0.557	0.799	0.697	0.632	No
DHQase I	2EGZ	1	1.014	0.990	403.0	0.604	0.676	0.861	0.876	1.017	0.861	0.433	Yes
DHQase I	2EGZ	2	0.349	0.541	55.5	0.675	0.549	0.809	0.237	1.409	0.168	0.229	No
DHQase I	3L9C	1	1.072	1.085	325.7	0.449	0.825	1.045	1.332	1.114	1.196	0.307	Yes
DHQase I	3L9C	2	0.917	0.947	273.3	0.654	0.618	0.852	0.346	1.208	0.286	1.065	No
DHQase I	3L9C	3	0.773	0.771	97.7	0.608	0.61	0.855	1.105	0.729	1.515	3.303	No
DHQase I	3L9C	4	0.645	0.732	100.6	0.613	0.692	0.949	0.678	1.142	0.593	1.071	No
DHQase I	3L9C	5	0.599	0.647	149.2	0.770	0.597	0.758	0.271	0.967	0.280	2.075	No
DHQase I	3L9C	6	0.586	0.670	128.6	0.673	0.643	0.770	0.15	1.116	0.134	1.49	No
DHQase I	3L9C	7	0.515	0.568	104.4	0.828	0.529	0.688	0.121	0.974	0.124	2.083	No
DHQase I	3S42	1	0.683	0.754	109.1	0.649	0.675	0.893	0.352	1.126	0.313	1.108	No
DHQase I	3S42	2	0.764	0.964	86.4	0.391	0.942	1.236	0.984	1.489	0.661	0.247	Yes
DHQase I	3S42	3	0.617	0.691	125.5	0.631	0.72	0.944	0.476	1.036	0.459	1.186	No
DHQase I	3S42	4	0.755	0.784	55.9	0.470	0.739	1.151	2.378	0.751	3.166	1.251	No
DHQase I	3S42	5	0.443	0.592	61.1	0.719	0.564	0.747	0.019	1.311	0.014	0.569	No
DHQase I	4CNN	1	0.984	0.989	282.6	0.595	0.715	0.885	0.427	1.092	0.391	0.449	Yes
DHQase I	4CNN	2	0.937	0.929	145.8	0.541	0.713	0.942	0.631	0.886	0.712	1.124	No
DHQase I	4CNN	3	0.689	0.723	83.0	0.552	0.632	0.909	0.812	0.941	0.863	0.672	No
DHQase I	6H5G	1	0.828	0.923	118.0	0.568	0.743	0.96	0.429	1.29	0.333	0.634	Yes
DHQase I	6H5G	2	0.960	0.934	131.4	0.564	0.69	0.880	1.134	0.756	1.501	1.196	No
DHQase I	6H5G	3	0.696	0.777	108.0	0.500	0.75	1.063	0.738	1.113	0.663	0.684	No
DHQase I	6SFH	1	0.974	1.012	154.6	0.463	0.796	1.057	1.425	1.152	1.238	0.478	Yes
DHQase I	6SFH	2	0.771	0.813	203.0	0.701	0.617	0.789	0.242	1.132	0.214	0.638	No
DHQase I	6SFH	3	0.691	0.767	85.7	0.493	0.770	1.073	1.226	1.074	1.142	1.338	No
DHQase I	6SFH	4	0.584	0.658	116.9	0.706	0.617	0.782	0.408	1.098	0.372	0.357	No
DHQase I	6SFH	5	0.580	0.625	63.4	0.624	0.575	0.891	1.069	0.908	1.177	0.447	No
DHQase II	1GQO	1	0.982	1.087	177.3	0.361	0.834	1.115	0.425	1.392	0.305	0.299	Yes

Enzyme	PDB ID	Site	Dscore	SiteScore	Volume	Exposure	Enclosure	Contact	Phobic	Philic	Balance	Don/Acc	SBS
DHQase II	3LWZ	1	1.014	1.013	353.3	0.481	0.745	1.014	0.934	1.071	0.872	0.913	Yes
DHQase II	3LWZ	2	0.625	0.647	49.6	0.632	0.622	0.886	1.872	0.549	3.409	5.465	No
DHQase II	3LWZ	3	0.569	0.581	64.4	0.786	0.513	0.637	0.701	0.552	1.269	6.426	No
DHQase II	3LWZ	4	0.493	0.542	52.1	0.750	0.536	0.714	0.991	0.795	1.246	4.700	No
DHQase II	4KIW	1	0.927	0.936	175.6	0.497	0.751	0.999	1.031	1.003	1.028	0.434	Yes
DHQase II	6HSA	1	0.683	0.962	82.0	0.304	0.925	1.324	0.458	1.747	0.262	0.15	Yes
DHQase II	6HSQ	1	0.980	1.009	284.0	0.571	0.711	0.94	0.242	1.186	0.204	0.438	Yes
DHQase II	6HSQ	2	0.514	0.562	56.9	0.758	0.595	0.784	0.663	0.671	0.989	0.849	No
DHQase II	6HSQ	3	0.427	0.529	40.8	0.612	0.612	1.006	0.482	1.074	0.449	1.185	No
DHQase II	6SME	1	1.032	1.039	231.5	0.422	0.795	1.048	1.399	1.078	1.298	0.37	Yes
DHQase II	6SME	2	0.580	0.614	72.8	0.652	0.547	0.78	0.962	0.833	1.156	1.797	No
SDH	3FBT	1	1.028	1.008	683.5	0.624	0.705	0.857	0.649	1.026	0.633	0.668	Yes
SDH	3FBT	2	0.662	0.689	101.3	0.667	0.567	0.794	0.510	0.948	0.537	1.104	No
SDH	3FBT	3	0.62	0.764	101.4	0.505	0.674	1.052	0.566	1.359	0.417	1.636	No
SDH	3FBT	4	0.635	0.671	154.0	0.766	0.564	0.761	0.308	1.003	0.307	1.351	No
SDH	3FBT	5	0.541	0.613	100.9	0.713	0.637	0.814	0.29	1.012	0.287	1.942	No
SDH	3O8Q	1	1.043	1.032	535.8	0.522	0.746	0.961	0.481	1.057	0.455	0.525	Yes
SDH	3O8Q	2	0.911	0.957	219.5	0.591	0.656	0.896	0.435	1.238	0.352	0.549	No
SDH	3O8Q	3	0.633	0.765	138.2	0.643	0.699	0.974	0.176	1.301	0.135	0.643	No
SDH	3O8Q	4	0.638	0.665	92.6	0.697	0.556	0.817	0.419	0.889	0.471	0.929	No
SDH	3O8Q	5	0.560	0.605	99.5	0.802	0.588	0.668	0.185	0.806	0.229	0.946	No
SDH	3O8Q	6	0.602	0.627	56.9	0.703	0.568	0.757	1.194	0.695	1.717	1.546	No
SDH	3TNL	1	1.032	1.028	895.7	0.556	0.740	0.978	0.812	1.079	0.753	0.682	Yes
SDH	3TNL	2	0.935	0.896	221.4	0.689	0.599	0.838	0.901	0.748	1.204	1.129	No
SDH	3TNL	3	0.798	0.799	136.8	0.655	0.612	0.769	0.584	0.875	0.667	0.592	No
SDH	4OMU	1	0.977	0.981	700.0	0.627	0.67	0.850	0.382	1.118	0.342	0.739	Yes
SDH	4OMU	2	0.945	0.916	188.9	0.635	0.628	0.898	0.992	0.854	1.162	0.802	No
SDH	4OMU	3	0.712	0.743	175.3	0.745	0.638	0.797	0.474	0.968	0.49	1.227	No
SDH	4OMU	4	0.584	0.620	72.0	0.729	0.568	0.728	0.711	0.807	0.881	6.336	No
SDH	4OMU	5	0.504	0.586	81.0	0.662	0.628	0.771	0.372	1.051	0.354	0.332	No
SDH	4OMU	6	0.518	0.555	68.8	0.759	0.53	0.623	0.406	0.707	0.574	0.903	No
SDH	4P4G	1	0.991	1.064	507.0	0.439	0.794	1.079	0.66	1.306	0.506	0.485	Yes

Enzyme	PDB ID	Site	Dscore	SiteScore	Volume	Exposure	Enclosure	Contact	Phobic	Philic	Balance	Don/Acc	SBS
SDH	4P4G	2	0.732	0.759	124.6	0.602	0.657	0.866	1.112	0.897	1.239	1.163	No
SDH	4P4G	3	0.459	0.524	59.9	0.708	0.556	0.726	0.452	0.871	0.519	1.443	No
SDH	4P4N	1	1.026	1.024	510.7	0.531	0.733	0.923	0.677	1.086	0.623	0.552	Yes
SDH	4P4N	2	0.787	0.820	150.1	0.486	0.698	1.004	1.218	1.046	1.164	0.987	No
EPSPS	1Q36	1	0.848	1.188	216.2	0.376	0.98	1.233	0.169	2.086	0.081	0.198	Yes
EPSPS	1Q36	2	0.628	0.696	129.3	0.701	0.613	0.804	0.190	1.116	0.171	1.276	No
EPSPS	1Q36	3	0.631	0.740	146.1	0.648	0.678	0.853	0.214	1.224	0.175	1.306	No
EPSPS	1Q36	4	0.638	0.681	105.3	0.695	0.661	0.875	0.915	0.812	1.127	1.342	No
EPSPS	1Q36	5	0.660	0.670	70.1	0.736	0.585	0.82	1.404	0.607	2.313	2.115	No
EPSPS	1Q36	6	0.419	0.578	102.5	0.777	0.638	0.864	0.079	1.272	0.062	1.247	No
EPSPS	1X8T	1	1.026	1.189	213.3	0.331	0.981	1.263	1.226	1.54	0.796	0.246	Yes
EPSPS	1X8T	2	0.690	0.801	155.2	0.563	0.684	0.961	0.350	1.281	0.273	1.012	No
EPSPS	1X8T	3	0.505	0.607	114.4	0.767	0.606	0.73	0.02	1.146	0.017	1.647	No
EPSPS	1X8T	4	0.594	0.650	97.1	0.724	0.672	0.94	1.081	0.826	1.308	3.485	No
EPSPS	1X8T	5	0.489	0.549	63.2	0.800	0.555	0.665	0.112	0.905	0.123	0.889	No
EPSPS	2AA9	1	0.693	1.132	144.1	0.370	0.98	1.259	0.164	2.338	0.07	0.213	Yes
EPSPS	2AA9	2	0.639	0.685	134.1	0.733	0.604	0.766	0.183	1.042	0.176	0.983	No
EPSPS	2AA9	3	0.665	0.686	126.9	0.766	0.586	0.761	0.486	0.792	0.613	2.980	No
EPSPS	2AA9	4	0.585	0.658	140.0	0.742	0.673	0.842	0.258	1.051	0.245	0.811	No
EPSPS	2AA9	5	0.503	0.591	93.8	0.788	0.592	0.74	0.094	1.1	0.085	1.828	No
EPSPS	2GG4	1	0.978	0.983	726.1	0.663	0.672	0.847	0.364	1.12	0.325	1.098	Yes
EPSPS	2GG4	2	0.723	0.769	76.7	0.577	0.711	0.932	1.562	1.002	1.558	0.390	No
EPSPS	2GG4	3	0.513	0.617	77.0	0.688	0.620	0.941	0.146	1.147	0.127	2.040	No
EPSPS	2GG6	1	0.852	1.103	286.4	0.383	0.852	1.104	0.344	1.838	0.187	0.643	Yes
EPSPS	2GG6	2	0.881	0.871	127.3	0.569	0.692	0.961	1.453	0.729	1.995	3.075	No
EPSPS	2GG6	3	0.708	0.840	132.1	0.482	0.727	1.133	0.441	1.344	0.328	1.372	No
EPSPS	2GG6	4	0.675	0.710	114.4	0.646	0.577	0.801	0.179	1.052	0.170	1.190	No
EPSPS	2GG6	5	0.706	0.742	183.8	0.713	0.632	0.808	0.283	1.038	0.273	1.362	No
EPSPS	2GG6	6	0.626	0.653	31.2	0.45	0.635	1.036	1.883	0.601	3.134	5.51	No
EPSPS	2GG6	7	0.622	0.627	66.2	0.763	0.559	0.595	0.896	0.475	1.888	1.525	No
EPSPS	3FJX	1	0.844	1.179	195.1	0.401	0.983	1.356	0.400	2.060	0.194	0.317	Yes
EPSPS	3FJX	2	0.769	0.811	155.6	0.702	0.588	0.718	0.275	1.155	0.238	0.807	No

Enzyme	PDB ID	Site	Dscore	SiteScore	Volume	Exposure	Enclosure	Contact	Phobic	Philic	Balance	Don/Acc	SBS
EPSPS	3FJX	3	0.608	0.677	139.3	0.715	0.644	0.796	0.202	1.075	0.188	1.227	No
EPSPS	3FJX	4	0.551	0.630	101.4	0.674	0.641	0.853	0.451	1.068	0.422	1.959	No
EPSPS	3FJX	5	0.462	0.557	96.9	0.809	0.572	0.708	0.049	1.108	0.044	1.277	No
SK	1L4U	1	0.902	1.043	422.9	0.462	0.762	1.068	0.4056	1.519	0.266	0.536	Yes
SK	1VIA	1	0.966	1.005	240.4	0.557	0.786	0.951	0.678	1.156	0.586	0.347	Yes
SK	1VIA	2	0.744	0.788	112.5	0.462	0.741	1.032	1.361	0.951	1.431	0.698	No
SK	1VIA	3	0.512	0.606	106.7	0.752	0.657	0.910	0.207	1.0789	0.1916	1.171	No
SK	1VIA	4	0.450	0.564	63.5	0.7101	0.600	0.903	0.208	0.861	0.241	1.643	No
SK	2IYQ	1	0.935	1.061	316.6	0.399	0.789	1.110	0.492	1.467	0.335	0.586	Yes
SK	2IYV	1	0.534	0.709	96.0	0.704	0.623	0.856	0.213	1.444	0.147	0.427	No
SK	2IYV	2	0.681	0.665	73.7	0.791	0.529	0.667	0.972	0.436	2.229	1.288	Yes
SK	2IYX	1	0.921	1.033	406.1	0.486	0.748	0.961	0.291	1.434	0.203	0.414	Yes
SK	3VAA	1	0.953	0.980	485.3	0.652	0.667	0.838	0.282	1.190	0.237	0.387	Yes
CS	1QXO	1	1.044	1.017	790.1	0.601	0.705	0.885	1.050	0.978	1.074	0.719	Yes
CS	1QXO	2	1.030	0.978	331.3	0.723	0.651	0.724	0.893	0.713	1.251	1.231	No
CS	1QXO	3	0.882	0.869	255.7	0.724	0.635	0.765	0.417	0.858	0.486	4.331	No
CS	1QXO	4	0.724	0.908	147.4	0.500	0.739	1.063	0.374	1.553	0.241	0.507	No
CS	1QXO	5	0.754	0.760	150.7	0.715	0.613	0.767	0.620	0.793	0.783	1.432	No
CS	1QXO	6	0.626	0.711	216.3	0.673	0.732	0.889	0.544	1.084	0.502	0.767	No
CS	1QXO	7	0.653	0.676	131.3	0.793	0.619	0.710	0.839	0.689	1.218	2.552	No
CS	1QXO	8	0.328	0.611	60.3	0.569	0.665	1.057	0.290	1.660	0.174	0.592	No
CS	1QXO	9	0.470	0.544	50.6	0.658	0.553	0.808	0.182	1.045	0.175	0.931	No
CS	1UM0	1	0.992	1.005	1847.7	0.561	0.705	0.908	0.693	1.139	0.609	0.830	Yes
CS	1UM0	2	0.975	1.010	200.6	0.481	0.724	0.911	0.597	1.195	0.499	1.365	No
CS	1UM0	3	0.927	0.927	152.5	0.579	0.708	0.923	0.971	0.990	0.981	1.415	No
CS	1UM0	4	0.828	0.820	198.7	0.695	0.636	0.781	0.819	0.750	1.092	0.753	No
CS	1UM0	5	0.768	0.909	101.4	0.481	0.956	1.334	1.356	1.250	1.084	0.965	No
CS	1UM0	6	0.631	0.675	85.4	0.667	0.609	0.835	0.501	0.963	0.521	0.854	No
CS	1UM0	7	0.663	0.694	78.9	0.714	0.629	0.774	1.341	0.806	1.665	0.743	No
CS	2G85	1	0.893	1.094	657.7	0.417	0.839	1.144	0.502	1.689	0.297	0.515	Yes
CS	2G85	2	0.991	0.950	196.5	0.676	0.623	0.823	0.765	0.826	0.926	0.673	No
CS	2G85	3	0.762	0.771	198.0	0.754	0.588	0.720	0.305	0.942	0.324	1.189	No

Enzyme	PDB ID	Site	Dscore	SiteScore	Volume	Exposure	Enclosure	Contact	Phobic	Philic	Balance	Don/Acc	SBS
CS	2G85	4	0.591	0.663	88.5	0.622	0.581	0.774	0.406	1.124	0.361	2.062	No
CS	2G85	5	0.608	0.631	58.9	0.686	0.571	0.755	0.629	0.686	0.917	1.786	No
CS	2G85	6	0.713	0.719	132.2	0.804	0.589	0.617	0.636	0.720	0.883	0.783	No
CS	2G85	7	0.529	0.624	97.3	0.690	0.622	0.898	0.359	1.127	0.318	0.792	No
CS	2G85	8	0.688	0.681	110.6	0.812	0.569	0.676	0.996	0.490	2.034	1.204	No
CS	2O11	1	1.017	1.035	820.8	0.483	0.751	0.983	0.914	1.145	0.798	0.746	Yes
CS	2O11	2	0.813	0.822	121.6	0.532	0.655	0.952	0.956	0.910	1.051	0.796	No
CS	2O11	3	0.707	0.757	190.5	0.740	0.626	0.811	0.239	1.101	0.217	1.218	No
CS	2O11	4	0.682	0.698	120.0	0.795	0.566	0.713	0.450	0.843	0.593	1.262	No
CS	2O11	5	0.583	0.652	89.7	0.664	0.564	0.782	0.436	1.117	0.390	1.140	No
CS	2O11	6	0.612	0.679	140.6	0.762	0.629	0.790	0.338	1.082	0.312	0.466	No
CS	2O11	7	0.481	0.578	70.8	0.645	0.650	0.870	0.232	1.073	0.216	1.322	No
CS	2O12	1	1.066	1.062	643.4	0.476	0.790	0.998	1.272	1.069	1.190	0.546	Yes
CS	2O12	2	0.962	0.947	111.3	0.489	0.728	0.960	2.087	0.799	2.611	0.282	No
CS	2O12	3	0.788	0.795	108.9	0.602	0.642	0.874	0.755	0.823	0.917	0.796	No
CS	2O12	4	0.649	0.757	189.8	0.722	0.663	0.904	0.252	1.248	0.202	1.202	No
CS	2O12	5	0.755	0.742	153.7	0.826	0.570	0.618	0.746	0.595	1.253	1.207	No
CS	2O12	6	0.578	0.619	87.9	0.702	0.543	0.692	0.392	0.957	0.409	1.556	No
CS	2O12	7	0.485	0.538	48.1	0.758	0.531	0.765	0.309	0.842	0.367	2.006	No
CS	2O12	8	0.624	0.681	137.8	0.764	0.633	0.777	0.424	1.053	0.403	0.483	No
CS	2QHF	1	0.930	1.064	485.3	0.467	0.794	1.053	0.510	1.492	0.342	0.554	Yes
CS	2QHF	2	0.964	0.932	213.0	0.648	0.585	0.776	0.507	1.003	0.506	0.860	No
CS	2QHF	3	0.674	0.893	86.5	0.433	0.851	1.268	1.633	1.558	1.048	0.328	No
CS	2QHF	4	0.720	0.817	198.8	0.688	0.650	0.843	0.167	1.277	0.131	1.367	No
CS	2QHF	5	0.740	0.752	185.5	0.805	0.567	0.645	0.122	0.988	0.124	1.157	No
CS	2QHF	6	0.679	0.702	141.1	0.811	0.570	0.701	0.262	0.921	0.284	1.280	No
CS	2QHF	7	0.583	0.9359	62.4	0.410	0.978	1.403	0.967	1.902	0.509	0.293	No
CS	2QHF	8	0.702	0.708	100.7	0.728	0.588	0.709	1.073	0.684	1.569	0.917	No
CS	2QHF	9	0.545	0.652	134.7	0.779	0.645	0.796	0.257	1.172	0.219	0.449	No

2.9.2. Graphical representation of druggability analyses

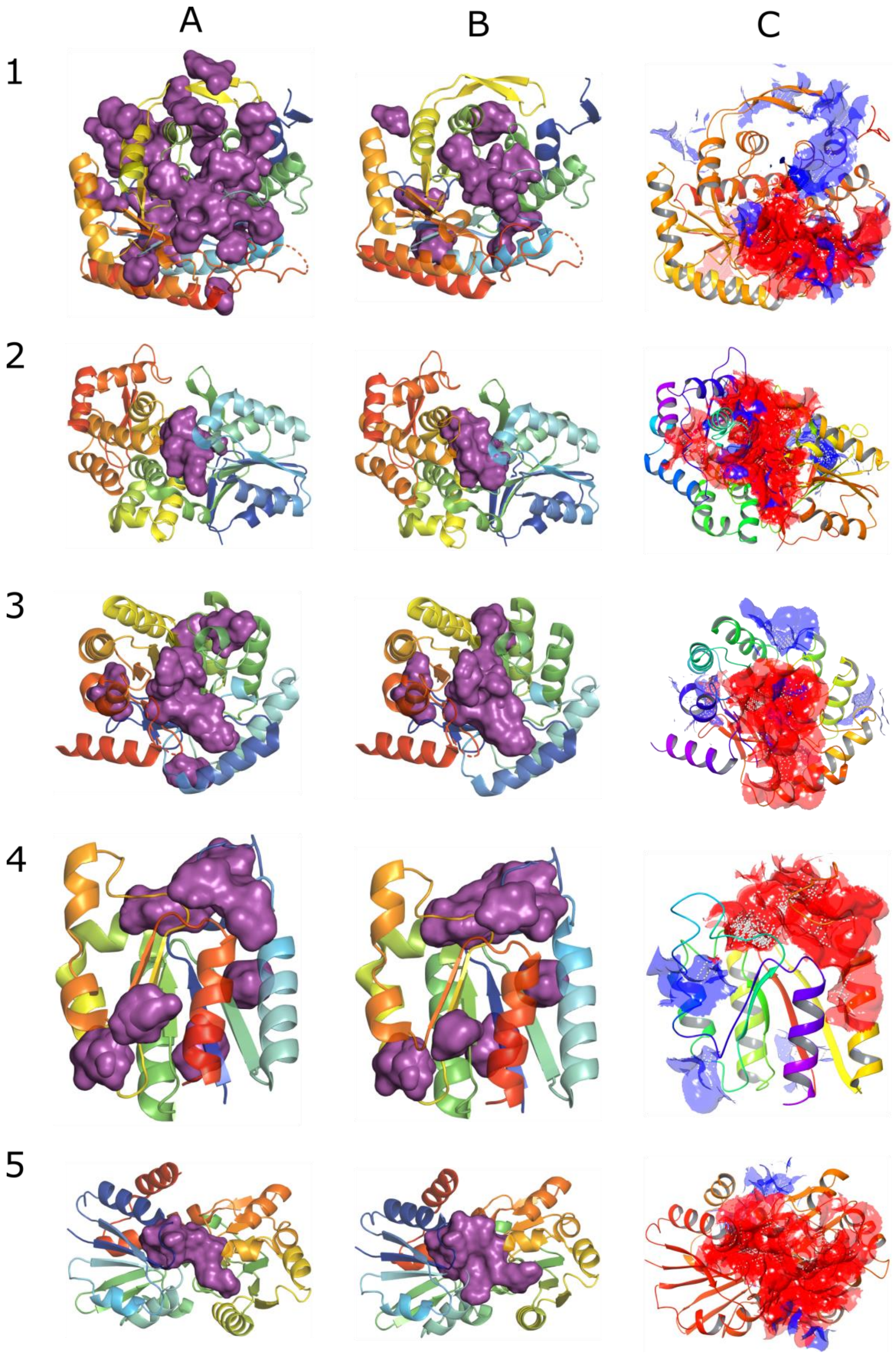

Figure S6. Surface representation of heatmaps and binding sites from structures of 1. DAHPS, 2. DHQS , 3. DHQase I, 4. DHQase II, identified by (**a**) FTMap, (**b**) FTSite and (**c**) Sitemap mapped onto X-ray structure of each enzyme in the shikimate pathway. PDB ID codes of enzymes are the same as in Figure 3 of the main manuscript. (**a**) tree top-ranked heatmaps are presented, (**b**) all binding sites are presented (**c**) two top-ranked binding sites are presented. Clusters in (**a**) and (**b**) are colored purple and sites in (**c**) are colored by their DScore in the following pattern: red(best ranked), blue(second best).

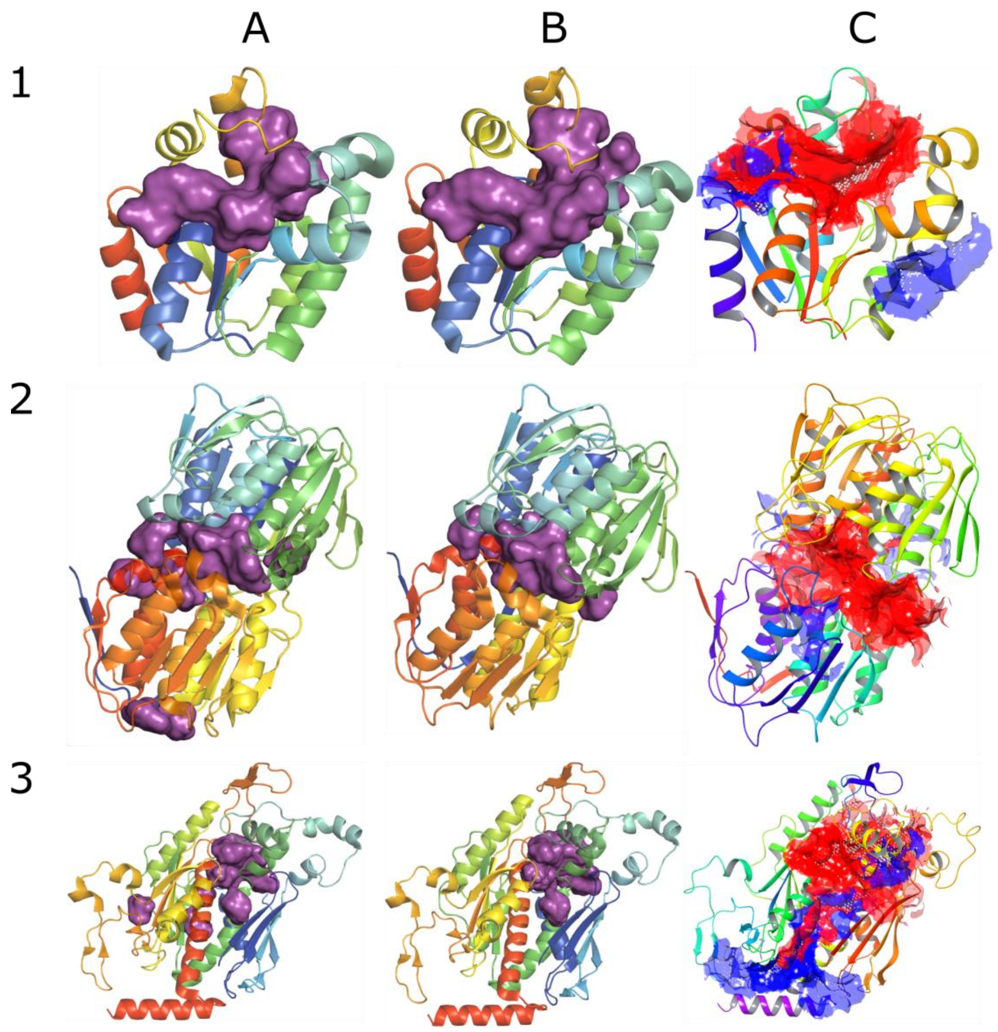

Figure S7. Surface representation of heatmaps and binding sites from structures of 1. SDH, 2. SK II, 3. EPSP and 4. CS identified by (**a**) FTMap, (**b**) FTSite and (**c**) Sitemap mapped onto X-ray structure of each enzyme in the shikimate pathway. PDB ID codes of enzymes are the same as in Figure 3 of the main manuscript. A) tree top-ranked heatmaps are presented, A) all binding sites are presented c) two top-ranked binding sites are presented. Clusters in (**a**) and (**b**) are colored purple and sites in (**c**) are colored by their DScore in the following pattern: red(best ranked), blue(second best).

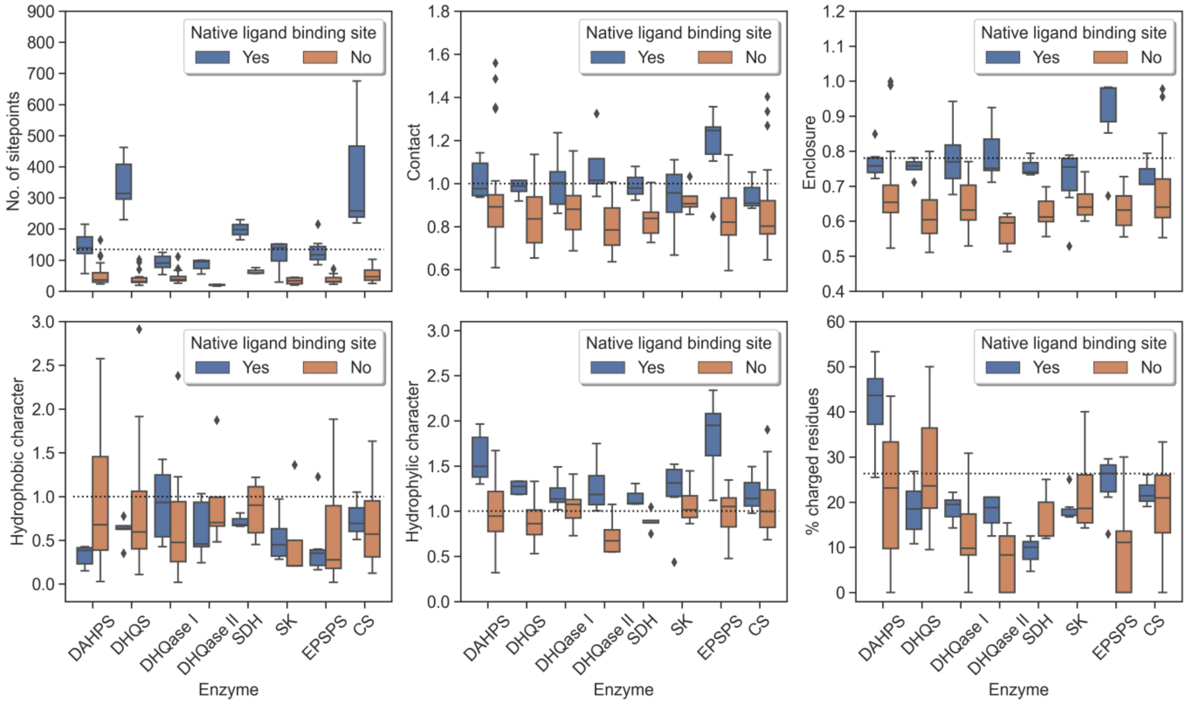

Figure S8. Differences in important physicochemical properties of the substrate and the allosteric binding sites for each enzyme of the shikimate pathway. A dotted line in each boxplot indicates a reference value for the average submicromolar inhibitor.

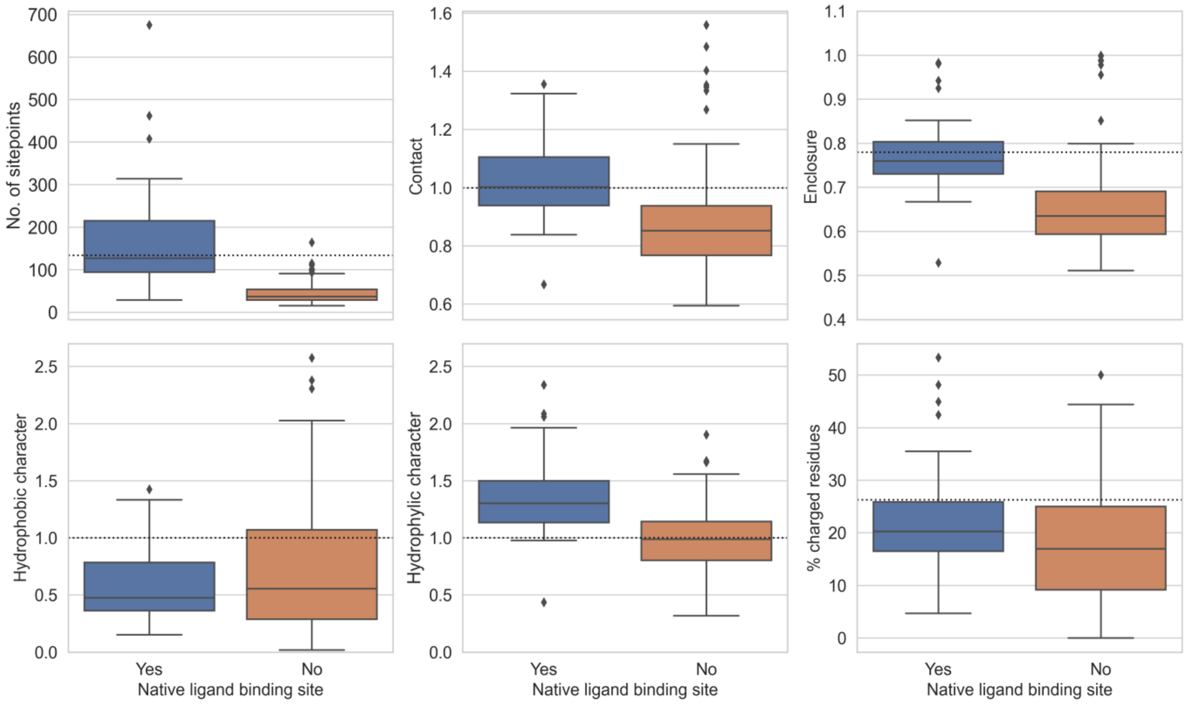

Figure S9. Differences in important physicochemical properties between the substrate and the allosteric binding sites. A dotted line in each boxplot indicates a reference value for the average submicromolar inhibitor.

3. References

1. Blanco, B.; Prado, V.; Lence, E.; Otero, J.M.; Garcia-Doval, C.; van Raaij, M.J.; Llamas-Saiz, A.L.; Lamb, H.; Hawkins, A.R.; Gonzalez-Bello, C. Mycobacterium Tuberculosis Shikimate Kinase Inhibitors: Design and Simulation Studies of the Catalytic Turnover. *J. Am. Chem. Soc.* **2013**, *135*, 12366–12376, doi:10.1021/ja405853p.
2. Balachandran, N.; Heimhalt, M.; Liuni, P.; To, F.; Wilson, D.J.; Junop, M.S.; Berti, P.J. Potent Inhibition of 3-Deoxy-D-Arabinoheptulosonate-7-Phosphate (DAHP) Synthase by DAHP Oxime, a Phosphate Group Mimic. *Biochemistry* **2016**, *55*, 6617–6629, doi:10.1021/acs.biochem.6b00930.
3. Walker, S.R.; Jiao, W.; Parker, E.J. Synthesis and Evaluation of Dual Site Inhibitors of 3-Deoxy-D-Arabino-Heptulosonate 7-Phosphate Synthase. *Bioorg. Med. Chem. Lett.* **2011**, *21*, 5092–5097, doi:10.1016/j.bmcl.2011.03.071.
4. Peterson, M.L.; Corey, S.D.; Font, J.L.; Walker, M.C.; Sikorski, J.A. New Simplified Inhibitors of EPSP Synthase: The Importance of Ring Size for Recognition at the Shikimate 3-Phosphate Site. *Bioorg. Med. Chem. Lett.* **1996**, *6*, 2853–2858, doi:10.1016/S0960-894X(96)00527-6.
5. Zhu, N.; Wang, X.; Li, D.; Lin, Y.; You, X.; Jiang, J.; Xu, Y.; Jiang, W.; Si, S. IMB-T130 Targets 3-Dehydroquinate Synthase and Inhibits Mycobacterium Tuberculosis. *Sci. Rep.* **2018**, *8*, 17439, doi:10.1038/s41598-018-35701-z.
6. Montchamp, J.L.; Frost, J.W. Cyclohexenyl and Cyclohexylidene Inhibitors of 3-Dehydroquinate Synthase: Active Site Interactions Relevant to Enzyme Mechanism and Inhibitor Design. *J. Am. Chem. Soc.* **1997**, *119*, 7645–7653, doi:10.1021/ja961771z.
7. Prado, V.; Lence, E.; Maneiro, M.; Vazquez-Ucha, J.C.; Beceiro, A.; Thompson, P.; Hawkins, A.R.; Gonzalez-Bello, C. Targeting the Motion of Shikimate Kinase: Development of Competitive Inhibitors That Stabilize an Inactive Open Conformation of the Enzyme. *J. Med. Chem.* **2016**, *59*, 5471–5487, doi:10.1021/acs.jmedchem.6b00483.
8. Gonzalez-Bello, C.; Manthey, M.K.; Harris, J.H.; Hawkins, A.R.; Coggins, J.R.; Abell, C. Synthesis of 2-Bromo- and 2-Fluoro-3-Dehydroshikimic Acids and 2-Bromo- and 2-Fluoroshikimic Acids Using Synthetic and Enzymatic Approaches. *J. Org. Chem.* **1998**, *63*, 1591–1597, doi:10.1021/jo971858i.
9. Lence, E.; Tizon, L.; Otero, J.M.; Peon, A.; Prazeres, V.F.V.; Llamas-Saiz, A.L.; Fox, G.C.; van Raaij, M.J.; Lamb, H.; Hawkins, A.R.; et al. Mechanistic Basis of the Inhibition of Type II Dehydroquinase by (2S)- and (2R)-2-Benzyl-3-Dehydroquinic Acids. *Acs Chem. Biol.* **2013**, *8*, 568–577, doi:10.1021/cb300493s.
10. Thomas, M.G.; Lawson, C.; Allanson, N.M.; Leslie, B.W.; Bottomley, J.R.; McBride, A.; Olusanya, O.A. A Series of 2(Z)-2-Benzylidene-6,7-Dihydroxybenzofuran-3[2H]-Ones as Inhibitors of Chorismate Synthase. *Bioorg. Med. Chem. Lett.* **2003**, *13*, 423–426, doi:10.1016/S0960-894X(02)00957-5.
11. Peek, J.; Shi, T.; Christendat, D. Identification of Novel Polyphenolic Inhibitors of Shikimate Dehydrogenase (AroE). *J. Biomol. Screen.* **2014**, *19*, 1090–1098, doi:10.1177/1087057114527127.
12. Bentley, R. The Shikimate Pathway - A Metabolic Tree with Many Branches. *Crit. Rev. Biochem. Mol. Biol.* **1990**, *25*, 307–384, doi:10.3109/10409239009090615.
13. Gibson, F.; Pittard, J. Pathways of Biosynthesis of Aromatic Amino Acids And Vitamins And Their Control In Microorganisms. *Bacteriol. Rev.* **1968**, *32*, 465-, doi:10.1128/MMBR.32.4_Pt_2.465-492.1968.
14. Davis, B. Aromatic Biosynthesis .1. The Role of Shikimic Acid. *J. Biol. Chem.* **1951**, *191*, 315–325.
15. Roberts, F.; Roberts, C.W.; Johnson, J.J.; Kyle, D.E.; Krell, T.; Coggins, J.R.; Coombs, G.H.; Milhous, W.K.; Tzipori, S.; Ferguson, D.J.P.; et al. Evidence for the Shikimate Pathway in Apicomplexan Parasites (Vol 393, Pg 801, 1998). *Nature* **1998**, *395*, 306–306, doi:10.1038/26277.
16. Herrmann, K.M.; Weaver, L.M. The Shikimate Pathway. *Annu. Rev. Plant Physiol. Plant Mol. Biol.* **1999**, *50*, 473–503, doi:10.1146/annurev.arplant.50.1.473.

17. Jiao, W.; Blackmore, N.J.; Nazmi, A.R.; Parker, E.J. Quaternary Structure Is an Essential Component That Contributes to the Sophisticated Allosteric Regulation Mechanism in a Key Enzyme from Mycobacterium Tuberculosis. *Plos One* **2017**, *12*, e0180052, doi:10.1371/journal.pone.0180052.
18. Jensen, R.A.; Xie, G.; Calhoun, D.H.; Bonner, C.A. The Correct Phylogenetic Relationship of KdsA (3-Deoxy-D-Manno-Octulosonate 8-Phosphate Synthase) with One of Two Independently Evolved Classes of AroA (3-Deoxy-D-Arabino-Heptulosonate 7-Phosphate Synthase). *J. Mol. Evol.* **2002**, *54*, 416–423, doi:10.1007/s00239-001-0031-z.
19. Webby, C.J.; Baker, H.M.; Lott, J.S.; Baker, E.N.; Parker, E.J. The Structure of 3-Deoxy-D-Arabino-Heptulosonate 7-Phosphate Synthase from Mycobacterium Tuberculosis Reveals a Common Catalytic Scaffold and Ancestry for Type I and Type II Enzymes. *J. Mol. Biol.* **2005**, *354*, 927–939, doi:10.1016/j.jmb.2005.09.093.
20. Light, S.H.; Anderson, W.F. The Diversity of Allosteric Controls at the Gateway to Aromatic Amino Acid Biosynthesis. *Protein Sci.* **2013**, *22*, 395–404, doi:10.1002/pro.2233.
21. Subramaniam, P.S.; Xie, G.; Xia, T.H.; Jensen, R.A. Substrate Ambiguity of 3-Deoxy-D-Manno-Octulosonate 8-Phosphate Synthase from Neisseria Gonorrhoeae in the Context of Its Membership in a Protein Family Containing a Subset of 3-Deoxy-D-Arabino-Heptulosonate 7-Phosphate Synthases. *J. Bacteriol.* **1998**, *180*, 119–127, doi:10.1128/JB.180.1.119-127.1998.
22. Sterritt, O.W.; Kessans, S.A.; Jameson, G.B.; Parker, E.J. A Pseudoisostructural Type II DAH7PS Enzyme from Pseudomonas Aeruginosa: Alternative Evolutionary Strategies to Control Shikimate Pathway Flux. *Biochemistry* **2018**, *57*, 2667–2678, doi:10.1021/acs.biochem.8b00082.
23. Harris, J.M.; GonzalezBello, C.; Kleanthous, C.; Hawkins, A.R.; Coggins, J.R.; Abell, C. Evidence from Kinetic Isotope Studies for an Enolate Intermediate in the Mechanism of Type II Dehydroquinases. *Biochem. J.* **1996**, *319*, 333–336, doi:10.1042/bj3190333.
24. Florova, G.; Denoya, C.D.; Morgenstern, M.R.; Skinner, D.D.; Reynolds, K.A. Cloning, Expression, and Characterization of a Type II 3-Dehydroquinate Dehydratase Gene from Streptomyces Hygroscopicus. *Arch. Biochem. Biophys.* **1998**, *350*, 298–306, doi:10.1006/abbi.1997.0536.
25. Romanowski, M.J.; Burley, S.K. Crystal Structure of the Escherichia Coli Shikimate Kinase I (AroK) That Confers Sensitivity to Mecillinam. *Proteins-Struct. Funct. Genet.* **2002**, *47*, 558–562, doi:10.1002/prot.10099.
26. UniProt Consortium UniProt: The Universal Protein Knowledgebase in 2021. *Nucleic Acids Res.* **2021**, *49*, D480–D489, doi:10.1093/nar/gkaa1100.
27. KEGG: Kyoto Encyclopedia of Genes and Genomes. Available online: http://www.kegg.jp/ (accessed on 14 October 2021).
28. Schrödinger Release 2021-3: Maestro; Schrödinger, LLC.: New York, NY, USA, 2021.
29. Kozakov, D.; Grove, L.E.; Hall, D.R.; Bohnuud, T.; Mottarella, S.; Luo, L.; Xia, B.; Beglov, D.; Vajda, S. The FTMap Family of Web Servers for Determining and Characterizing Ligand Binding Hot Spots of Proteins. *Nat. Protoc.* **2015**, *10*, 733–755, doi:10.1038/nprot.2015.043.
30. The PyMOL Molecular Graphics System, Version 2.3.3.; Schrödinger, LLC.: New York, NY, USA, 2019.
31. Gonçalves, W.R.S.; Gonçalves-Almeida, V.M.; Arruda, A.L.; Meira, W., Jr.; da Silveira, C.H.; Pires, D.E.V.; de Melo-Minardi, R.C. PDBest: A User–Friendly Platform for Manipulating and Enhancing Protein Structures. *Bioinformatics* **2015**, *31*, 2894–2896, doi:10.1093/bioinformatics/btv223.
32. KNIME: The Konstanz Information Miner | SpringerLink Available online: https://link.springer.com/chapter/10.1007/978-3-540-78246-9_38 (accessed on 21 June 2021).
33. Schrödinger Release 2021-3: SiteMap; Schrödinger, LLC, New York, NY., 2021.
34. Waskom, M.L. Seaborn: Statistical Data Visualization. *J. Open Source Softw.* **2021**, *6*, 3021, doi:10.21105/joss.03021.

35. Hunter, J.D. Matplotlib: A 2D Graphics Environment. *Comput. Sci. Eng.* **2007**, *9*, 90–95, doi:10.1109/MCSE.2007.55.
36. Kluyver, T.; Ragan-Kelley, B.; Perez, F.; Granger, B.; Bussonnier, M.; Frederic, J.; Kelley, K.; Hamrick, J.; Grout, J.; et al. Jupyter Notebooks – a Publishing Format for Reproducible Computational Workflows. *Position. Power Acad. Publ. Play. Agents Agendas* **2016**, 87–90, doi:10.3233/978-1-61499-649-1-87.
37. Ashkenazy, H.; Abadi, S.; Martz, E.; Chay, O.; Mayrose, I.; Pupko, T.; Ben-Tal, N. ConSurf 2016: An Improved Methodology to Estimate and Visualize Evolutionary Conservation in Macromolecules. *Nucleic Acids Res.* **2016**, *44*, W344-350, doi:10.1093/nar/gkw408.
